# The cell adhesion protein CAR is a negative regulator of synaptic transmission

**DOI:** 10.1038/s41598-019-43150-5

**Published:** 2019-05-01

**Authors:** Uta Wrackmeyer, Joanna Kaldrack, René Jüttner, Ulrike Pannasch, Niclas Gimber, Fabian Freiberg, Bettina Purfürst, Dagmar Kainmueller, Dietmar Schmitz, Volker Haucke, Fritz G. Rathjen, Michael Gotthardt

**Affiliations:** 1 Neuromuscular and Cardiovascular Cell Biology, Max Delbrück Center for Molecular Medicine, 13125 Berlin, Germany; 2 Neuroscience Research Center, Cluster of Excellence NeuroCure, Charité, 10117 Berlin, Germany; 3 Developmental Neurobiology, Max Delbrück Center for Molecular Medicine, 13125 Berlin, Germany; 4 Department of Molecular Pharmacology and Cell Biology, Leibniz Forschungsinstitut für Molekulare Pharmakologie (FMP), 13125 Berlin, Germany; 5 Core Facility Electron Microscopy, Max Delbrück Center for Molecular Medicine, 13125 Berlin, Germany; 6 Biomedical Image Analysis, Max Delbrück Center for Molecular Medicine and Berlin Institute of Health, 13125 Berlin, Germany

**Keywords:** Patch clamp, Molecular neuroscience, Synaptic vesicle exocytosis, Neurophysiology

## Abstract

The Coxsackievirus and adenovirus receptor (CAR) is essential for normal electrical conductance in the heart, but its role in the postnatal brain is largely unknown. Using brain specific CAR knockout mice (KO), we discovered an unexpected role of CAR in neuronal communication. This includes increased basic synaptic transmission at hippocampal Schaffer collaterals, resistance to fatigue, and enhanced long-term potentiation. Spontaneous neurotransmitter release and speed of endocytosis are increased in KOs, accompanied by increased expression of the exocytosis associated calcium sensor synaptotagmin 2. Using proximity proteomics and binding studies, we link CAR to the exocytosis machinery as it associates with syntenin and synaptobrevin/VAMP2 at the synapse. Increased synaptic function does not cause adverse effects in KO mice, as behavior and learning are unaffected. Thus, unlike the connexin-dependent suppression of atrioventricular conduction in the cardiac knockout, communication in the CAR deficient brain is improved, suggesting a role for CAR in presynaptic processes.

## Introduction

Synaptic transmission requires the formation and alignment of pre- and postsynaptic structures, which depends on diverse cell adhesion proteins^1^. During embryonic development, these proteins mediate neuronal migration and neurite outgrowth^2,3^. Their role in synaptic plasticity and transmission extends to the adult brain, where homo- or heterophilic interactions not only provide a mechanical link between neurons, but regulate signal transduction^4^. Cell adhesion proteins of the immunoglobulin superfamily (IgSF) contribute to synaptic differentiation and plasticity and are relevant to human disease^5–7^. They modulate the number of excitatory synapses^8^ or regulate presynaptic processes such as the mobilization and recycling of synaptic vesicles^9,10^.

The Coxsackievirus-adenovirus-receptor (CAR) is a member of the IgSF and has been initially identified as a virus receptor relevant to human myocarditis and encephalitis^11,12^. CAR is predominantly expressed in the developing heart and brain^13,14^ and crucial for prenatal development. Germline deletion of CAR affects cell-cell contact formation in the heart leading to intra-cardiac bleeding and embryonic lethality at E11.5^15,16^. In the adult heart, CAR is required for normal electrical conductance between atria and ventricle, which depends on the interaction of CAR with gap junction proteins^17,18^.

Although the perinatal regulation of CAR expression in the brain has been known since more than a decade^19^, its function in the central nervous system (CNS) is still largely elusive. Molecular and cell based studies have indicated a role of CAR in neuronal signaling: The two extracellular Ig-domains of CAR mediate homo- and heterodimerization and interact with extracellular matrix proteins, which affects neurite outgrowth *in vitro*^20^. Its cytoplasmic C-terminus contains a PDZ-binding motif, which mediates the interaction with PDZ (PSD-95/Disc-large/ZO-1)-domain containing proteins such as ZO-1, LNX and MAGI1, which link CAR to gap-junction proteins, the notch-signaling pathway, and the cytoskeleton^17,21–23^. Additional intracellular binding partners are the neuronal proteins PSD95 and Pick1, which also contain PDZ domains^24^. Pick1 interacts with the GluR2 subunit of the AMPAR and regulates the recycling of AMPAR to the cell surface^25,26^. PSD95 is a postsynaptic scaffold protein that interacts with the NMDAR and various cell adhesion proteins^27,28^ and affects synaptic transmission and long-term potentiation^29^. Recent functional studies in a CAR knockout with early embryonic depletion of CAR suggested a role in neurogenesis, synaptic function, and behavior^30^.

Based on the role of CAR in cardiac electro-conduction, the parallel perinatal regulation of CAR expression in heart and brain, and the available *in vitro* data on CAR’s neuronal functions^13,20,24^, we asked if the residual CAR expression in the adult brain is required for normal neuronal function, and which pathways might be affected by CAR *in vivo*.

In our brain specific knockout we used a Cre transgene that is not expressed during embryogenesis and barely detected in the early postnatal days to specifically study CAR in the postnatal brain. Our electrophysiological, histochemical, and biochemical analyses revealed that unlike the early depletion of CAR, which reduced synaptic function^30^, our postnatal CAR-knockout leads to improved synaptic transmission. Postnatally, CAR determines the strength of excitatory transmission via its interaction with the synaptic vesicle cycling machinery. Thus, we identify CAR as a negative regulator of neuronal communication with a critical role in presynaptic neurons and suggest that therapeutic inhibition of CAR in adults would enhance rather than suppress cognitive function.

## Results

### Increased synaptic transmission in brain specific CAR knockout mice

**Figure 1:**
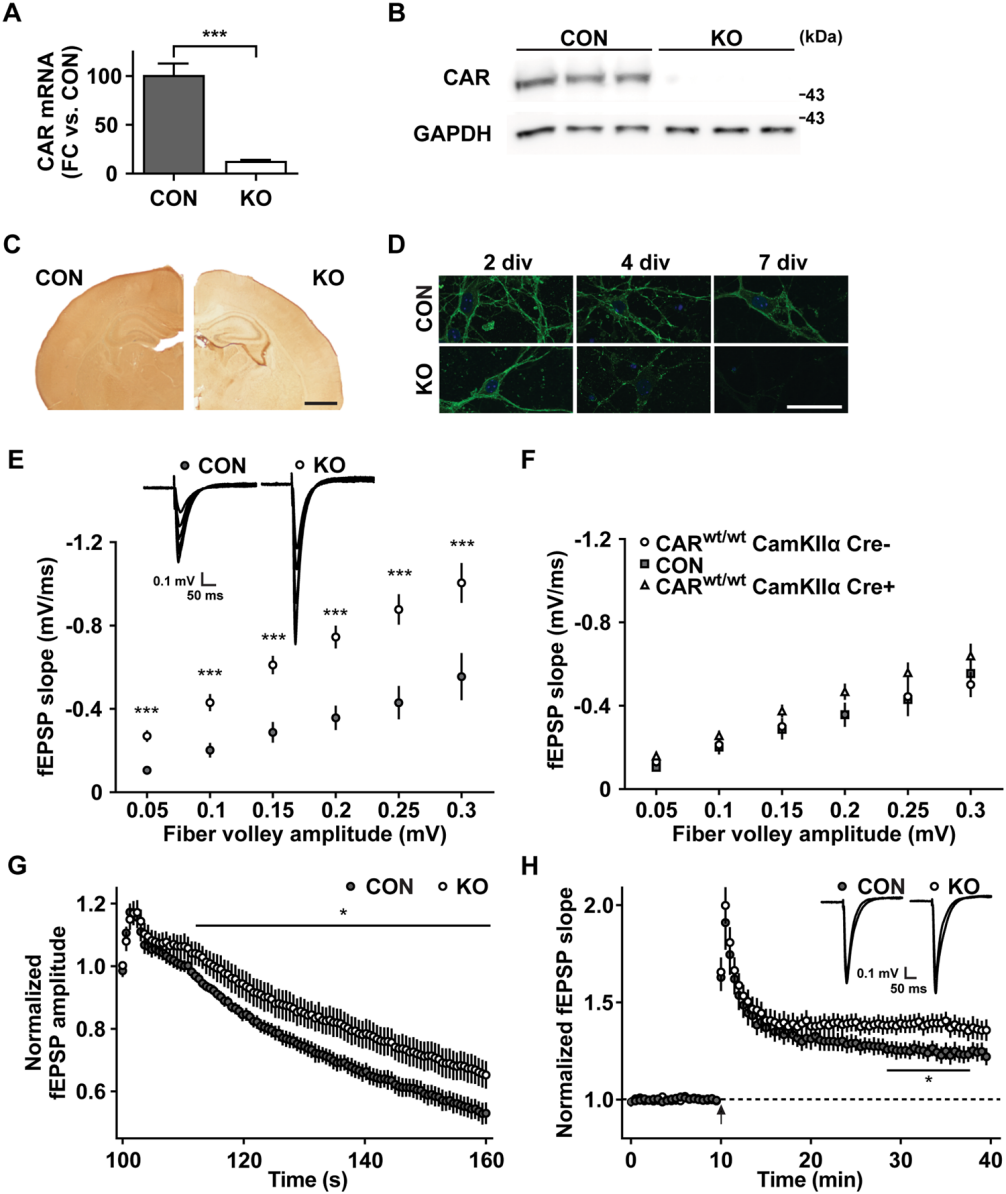
Increased synaptic transmission and plasticity in CAR KO mice. (**A**) qRT-PCR. At postnatal day 7 (P7) CAR mRNA-levels are reduced to 12% in knockout brains (KO) as compared to controls (CON) (FC, fold change). (**B**) In KO cerebrum CAR protein levels were below the detection limit of the Western-Blot at P7. GAPDH was used as a loading control. (**C**) Immunocytochemistry. Anti-CAR staining on P7 brain slices from KO and control animals (CON) demonstrate reduced CAR levels throughout the cerebrum (Scale bar, 1 mm). (**D**) Immunofluorescence of hippocampal neurons derived from two-day-old control and CAR KO mice (Scale bar, 10 µm). Staining with an anti-CAR antibody (green) demonstrates the reduction of CAR signal starting from 4 days *in vitro* (div). (**E**,**F**) Input-Output measurement on brain slices of three-month-old mice. (**E**) For each input (fiber volley amplitude), the output (fEPSP slope) is increased in CAR KO slices (CON, n = 10; KO, n = 10; ***p < 0.005). Representative traces of control and KO are illustrated above. (**F**) Input-Output responses of the appropriate control mice: CAR^wt/wt^ CamKIIαCre^−^ (n = 10), CAR^wt/wt^ CamKIIαCre^+^ (n = 9), and CAR^lox/lox^ CamKIIαCre^−^ (CON) (n = 10). (**G**) The response to prolonged repetitive stimulation (600 pulses at 10 Hz) declines less and more slowly in CAR KO mice compared to controls. Responses were normalized to fEPSP amplitudes before the onset of stimulation (CON, n = 8; KO, n = 8; *p < 0.05). (**H**) LTP, induced by tetanic stimulation of Schaffer collaterals (arrow, two 100 Hz tetani for 1 s, separated by 20 s) is slightly increased (*p < 0.05, 18–28 min after induction) in CAR KO mice (n = 12) compared to control slices (n = 10). Sample traces represent averaged fEPSPs before and 30 min after tetanization. Responses were binned (bin size 30 s) and normalized to fEPSP amplitudes measured before the onset of stimulation.

CAR is expressed in the pre- and neonatal brain, including neurons, astrocytes and microglia^14,31^. To study the neuronal functions of CAR we have generated a brain specific CAR knockout (KO) mouse using the cre-lox-recombination system, which circumvents the embryonic lethality of the conventional CAR knockout^15,16,18^. To direct CAR deficiency to the postnatal brain and reduce potentially complicating developmental effects, we used the CamKIIα-Cre, which is active from postnatal day P3^32^. Breeding floxed CAR Exon1 (CAR^lox/lox^) mice with CamKIIα-Cre transgenics resulted in viable brain specific KO mice (CAR^lox/lox^ CamKIIαCre^+^). Their phenotypic appearance, body weight, and reproduction capacity were normal with offspring delivered at the expected Mendelian ratios. Excision of exon 1, which contains the ATG is expected to eliminate expression of all CAR isoforms. Indeed, CAR mRNA levels were reduced ~10-fold in the 7-day-old brain specific knockout as compared to CAR^lox/lox^ CamKIIαCre^−^ control mice (CON), which carry the floxed allele but lack the cre transgene and therefore express normal levels of CAR (Fig. 1A). On the protein level, CAR expression was reduced to below the detection limit of the western blot in the cerebrum of 1-week-old mice (Fig. 1B) and affected almost all brain regions except the meninges, as revealed by immunohistochemical staining of brain slices (Fig. 1C). In neuronal cultures generated from two-day-old CAR KO mice the CAR signal decreased over time of cultivation starting from 4 days *in vitro* (4 div) and was undetectable at 7 div (Fig. 1D), which demonstrates an efficient deletion of CAR in this KO strain.

We and others have previously demonstrated that the heart specific deletion of CAR in adult mice leads to a cardiac conduction problem that manifests as an isolated atrioventricular block^17,18^. To determine if CAR is not only involved in intercellular communication in the heart but also in the brain we evoked AMPA receptor (AMPAR)-mediated field excitatory postsynaptic potentials (fEPSPs) in CA1 Schaffer collaterals of acute hippocampal slices of 3-month-old CAR KO and control mice. We compared the size of the presynaptic fiber volley (input) to the slope of the fEPSP (output) and found that synaptic transmission was significantly increased in CAR KO brain slices versus control animals (Fig. 1E). To exclude an effect introduced by the intronic lox-sites or the CamKIIα-Cre transgene, we repeated the fEPSP measurements on wild-type animals (CAR^wt/wt^ CamKIIαCre^−^) and CAR^wt/wt^ CamKIIαCre^+^ mice (Fig. 1F). As neither strain behaved differently from the floxed control mice we used CAR^lox/lox^ CamKIIαCre^−^ as a control for all future experiments.

Next, we measured the response of CAR KO and control mice to prolonged repetitive stimulation of the neurons in the CA1 region. The prolonged electrically stimulation leads to depletion of the vesicle pool and saturation of neurotransmitter receptors and thus to a decrease in fEPSP amplitude over time. Unexpectedly, the synaptic response of CAR KO pyramidal neurons declined more slowly and depressed less as compared to neurons from control mice, indicating a reduced synaptic fatigue during high-frequency stimulation (Fig. 1G). To determine if the altered synaptic fatigue has an effect on plasticity we induced long-term potentiation (LTP) in CAR KO and control mice by brief tetanic stimulation of Schaffer collaterals. The recorded field potentials were significantly increased in KO mice compared to controls at later time-points, representing an enhanced LTP in the KO (Fig. 1H).

### CAR deficiency affects presynaptic morphology and function

**Figure 2:**
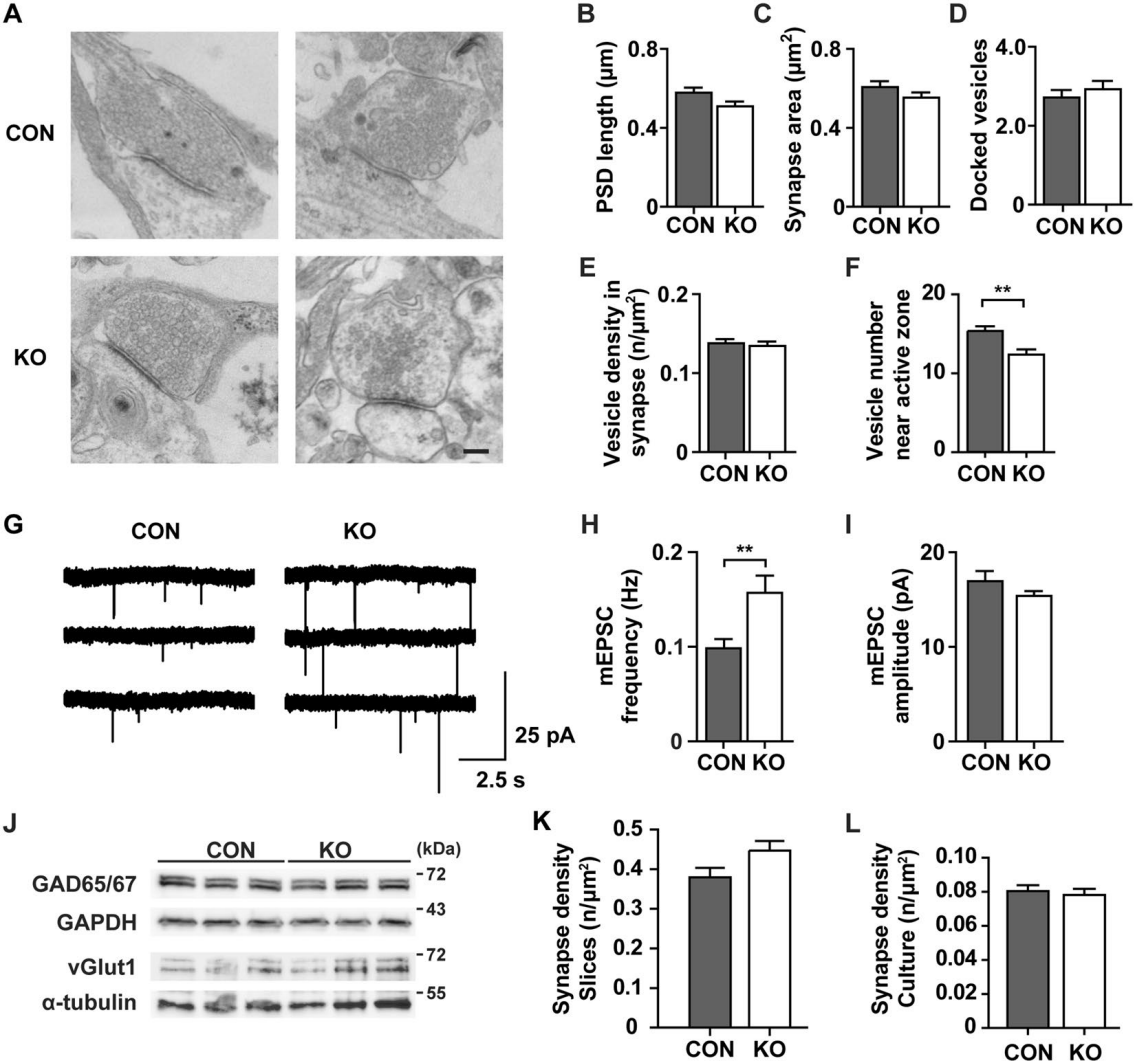
CAR KO mice have increased excitatory synaptic transmission. (**A**–**F**) Electron microscopy of isolated hippocampal neurons (13/14 div). (**A**) Representative pictures of control and CAR KO synapses (Scale bar, 200 nm). (**B**–**F**) Quantitative comparison of morphological parameters from control and CAR KO hippocampal neurons. The length of the postsynaptic density (PSD), the area of the presynapse, the number of docked vesicles and the density of vesicles in the presynapse were unchanged between genotypes. However, CAR KO mice have a significantly reduced (**p < 0.01) number of vesicles near the active zone (CON, n = 48; KO, n = 67 synapses). (**G**–**I**) Patch clamp recordings (CON, n = 24; KO, n = 25) in brain slices (postnatal day 24–28). (**G**) Example traces of mEPSCs of control and CAR KO. (**H**) The mEPSC frequency in CAR KO brain slices is significantly increased (**p < 0.01) as compared to controls while the mEPSC amplitude (**I**) is unchanged. (**J**) Neuronal markers proteins representing inhibitory and excitatory neurons are expressed at similar levels in brains from 3-month-old control and CAR-KO mice. Blots were cropped from different gels. (**K**) The number of synapses were counted in hippocampal slice at postnatal day 26 for control and CAR KO mice (CON, n = 4; KO, n = 10). (**L**) Synapse density in hippocampal cell cultures (CON: 8 coverslips; KO: 7 coverslips).

Since increased LTP suggests a synaptic function of CAR, we went on to study the ultrastructure of CAR KO synapses in electron microscopy. We found an unchanged PSD length, synapse area, number of docked vesicles and vesicle density; however, the number of vesicles near the active zone (within 150 nm from active zone) was reduced in the CAR KO (Fig. 2A–F).

In order to further investigate the presynaptic effect of CAR deletion, we measured miniature excitatory postsynaptic currents (mEPSC). In hippocampal slices from ~3.5 week-old CAR KO mice the mEPSC frequency was increased compared to control animals, suggesting that CAR negatively regulates spontaneous neurotransmission (Fig. 2G,H). Of note, the mEPSC amplitude, a measure for postsynaptic neurotransmitter receptor strength, was unchanged in CAR KO versus control mice (Fig. 2I). The same applies to the rise and decay times of mEPSCs (Supplement Fig. 2A–C).

Basal excitatory synaptic transmission is in part determined by the ratio of excitatory to inhibitory signals^33,34^. To investigate whether CAR modulates the number of excitatory compared to inhibitory neurons we analyzed marker proteins for interneurons (glutamate decarboxylase 1 and 2, GAD 65/67) and excitatory neurons (the vesicular glutamate transporter, vGlut1). We did not find differences in expression levels of these proteins in CAR KO versus control mice (Fig. 2J). To evaluate if the increase in mEPSC frequency at postnatal day P26 results from increased synaptic density, we quantified synapses numbers in hippocampal slices at P26, as well as cell cultures at the matching day *in vitro*. In CAR deficient mice, synapse density was neither affected in hippocampal slices nor in hippocampal cell cultures (Fig. 2K,L). The density of synapses in hippocampal cell cultures was estimated manually, while the more complex analysis of synapse density in slices was estimated using an automated image analysis approach with machine learning. Neither our knockout nor the published knockdown^35^ had increased synaptic density with reduced CAR levels, suggesting a different mechanism underlying the increased mEPSC frequency.

### Synaptic vesicle endocytosis is facilitated in CAR KO neurons

**Figure 3:**
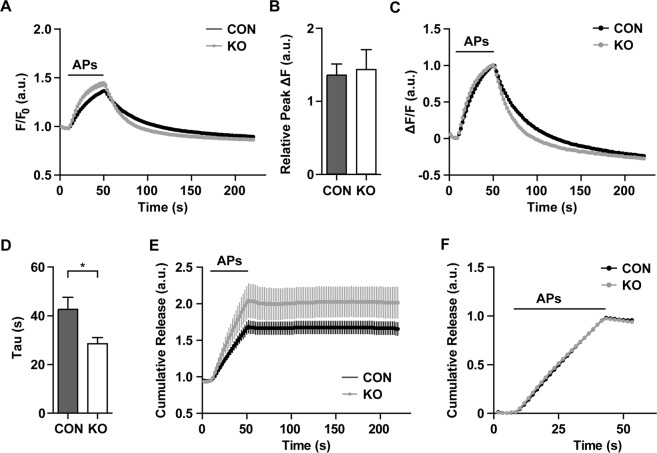
CAR depletion enhances synaptic vesicle endocytosis. (**A**) Average intensity-traces of DIV 14 hippocampal neurons expressing synaptophysin-pHluorin in response to 200 action potentials (APs) applied at 5 Hz. (**B**) Calculation of the relative peak of traces in (**A**). The peak fluorescence is unchanged in CAR KO in comparison to controls. However, changes in exocytosis might be covered by different rates of endocytosis. (**C**) Normalized average intensity-traces of (**A**). (**D**) Endocytic time constants (τ) of traces in (**C**). The speed of endocytosis is facilitated in CAR KO neurons when compared to controls (*p < 0.05). (**E**) Cumulative release rates were calculated by deconvolution of traces in (**A**) showing a slight increase in the amount of exocytosed vesicles in CAR knockout mice, that was covered in (**A**) by a faster endocytosis. (**F**) Normalization of the deconvolved traces in (**E**). The speed of exocytosis remains unaltered between CAR KO and control animals. KO, n = 14; CON, n = 13; 5 independent experiments; Data represent mean ± SEM; a.u. – arbitrary units.

The increased frequency of miniature EPSC led us to investigate if CAR might also affect evoked vesicle exocytosis and recycling. Here, we used live-cell imaging of cultured hippocampal neurons from CAR KO and control mice expressing synaptophysin-pHluorin, a chimera between the synaptic vesicle protein synaptophysin and pH-sensitive super ecliptic pHluorin (pH-sensitive GFP). PHluorin probes allow monitoring of exo-endocytosis due to their stimulation-induced fluorescence dequenching upon exocytosis and subsequent post-endocytic re-quenching as vesicles re-acidify. Neurons were stimulated with 200 action potentials (APs) at a frequency of 5 Hz and synaptic vesicle exo-endocytosis was monitored by following synaptophysin-pHluorin fluorescence. The relative peak fluorescence at the end of the stimulation period was similar in CAR knockout neurons in comparison to controls (Fig. 3A,B). However, since ongoing endocytosis during stimulation counteracts the accumulation of synaptic vesicle protein at the cell surface, synaptophysin-pHluorin peak fluorescence values cannot serve as a direct readout for exocytosis. In order to compensate for the possible confounding effects of endocytosis and to further characterize the role of CAR in regulating presynaptic exocytosis, we applied an established deconvolution routine^36^ that allows the determination of the total cumulated amount of exocytosis. Cumulative exocytosis tended to be increased in CAR KO neurons (Fig. 3E), although this difference did not reach statistical significance. Normalization of the peak fluorescence value to that observed in control neurons showed that the kinetics of exocytosis were unchanged in neurons from CAR KO mice (Fig. 3F), suggesting that CAR does not regulate the core release machinery itself. Interestingly, recycling synaptic vesicles from CAR KO neurons underwent significantly faster endocytosis and re-acidification (Fig. 3C,D), consistent with the partial resistance of slices from CAR KO mice to synaptic rundown and fatigue. These results suggest that loss of CAR is associated with a mild increase in stimulation-induced exocytosis and a significant facilitation of synaptic vesicle protein endocytosis.

### Normal behavior and learning in CAR KO mice

**Figure 4:**
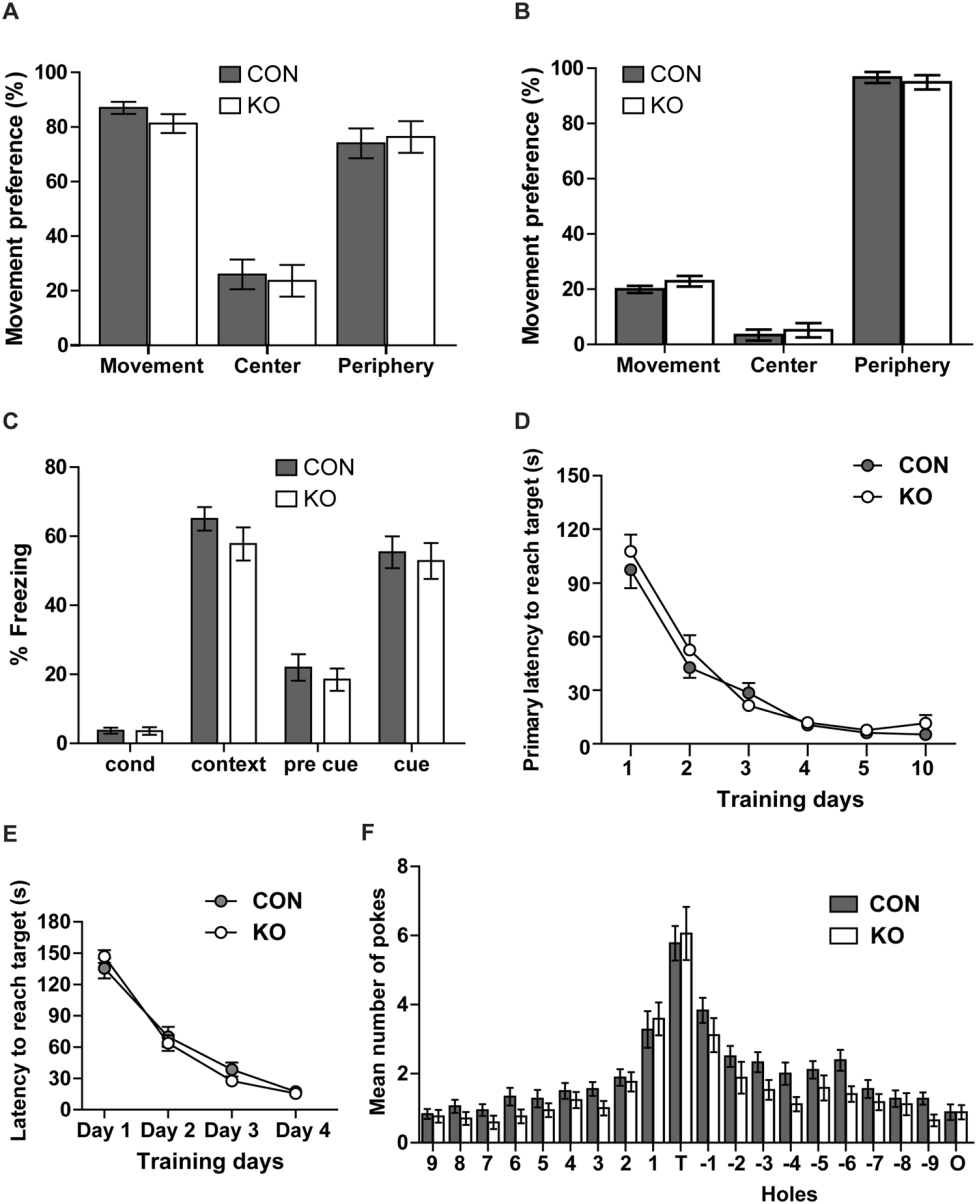
Normal behavior and learning in CAR KO mice. (**A**,**B**) Open field test. (**A**) Activity of adult CAR KO and control mice during the first 3 minutes after entering a new cage is similar between groups. Both knockout and control mice spend ~20% of their first 3 minutes in the center of the cage versus the periphery (CON, n = 7; KO, n = 14). (**B**) Activity of both groups with 24 h. (**C**) Context-fear-conditioning to test associated learning. Cue: conditioned stimulus (tone), context: unconditioned stimulus (foot shock). Recognition of unconditioned (context) and conditioned stimulus (sound) was similar in control and KO mice as detected by increase in freezing (CON, n = 18; KO, n = 17). (**D**) Barnes Maze as a test for spatial memory. Primary latency as parameters of learning decreased with time at similar rated in KO and control mice from training day 1 to 4 and the experimental days 5 and 10 (CON, n = 18; KO, n = 17). (**E**) Learning phase (day 1–4) of the Barnes maze test - control versus knockout mice. (**F**) Number of pokes into the target hole (T), holes 1 to 9 towards the left, holes −1 to −9 towards the right, and the opposite hole (O) are unchanged between KO and controls at day 5 (CON, n = 18; KO, n = 17).

LTP has been proposed to reflect the molecular processes that underlie learning and memory formation^37,38^. Especially the hippocampus plays a major role in associated and spatial learning^39,40^. We therefore analyzed the learning and memory performance in CAR KO compared to control mice. The basal exploration behavior in the Open-Field test was identical between both groups (Fig. 4A,B), which facilitated the analysis of the subsequent learning tests. We compared the associated and spatial learning ability of CAR KO versus control mice using context-fear-conditioning and the Barnes Maze tests (Fig. 4C–F). In the context-fear-conditioning experiment we compared the percentage of freezing of KO versus control mice as a parameter for fear (and thus recognition) and did not detect differences in the association of an unconditioned stimulus with a context or with a conditioned stimulus one day after conditioning (Fig. 4C). In the Barnes maze, the spatial learning test, both groups learned equally fast to find a hidden box (primary latency) and remembered similarly well the location of the target hole (Fig. 4D,F). Thus, although the synaptic transmission and LTP is increased in CAR KO mice it does not correlate with spatial and associated learning performance in our CAR KO model.

### CAR deficiency leads to upregulation of synaptotagmin 2 expression

**Figure 5:**
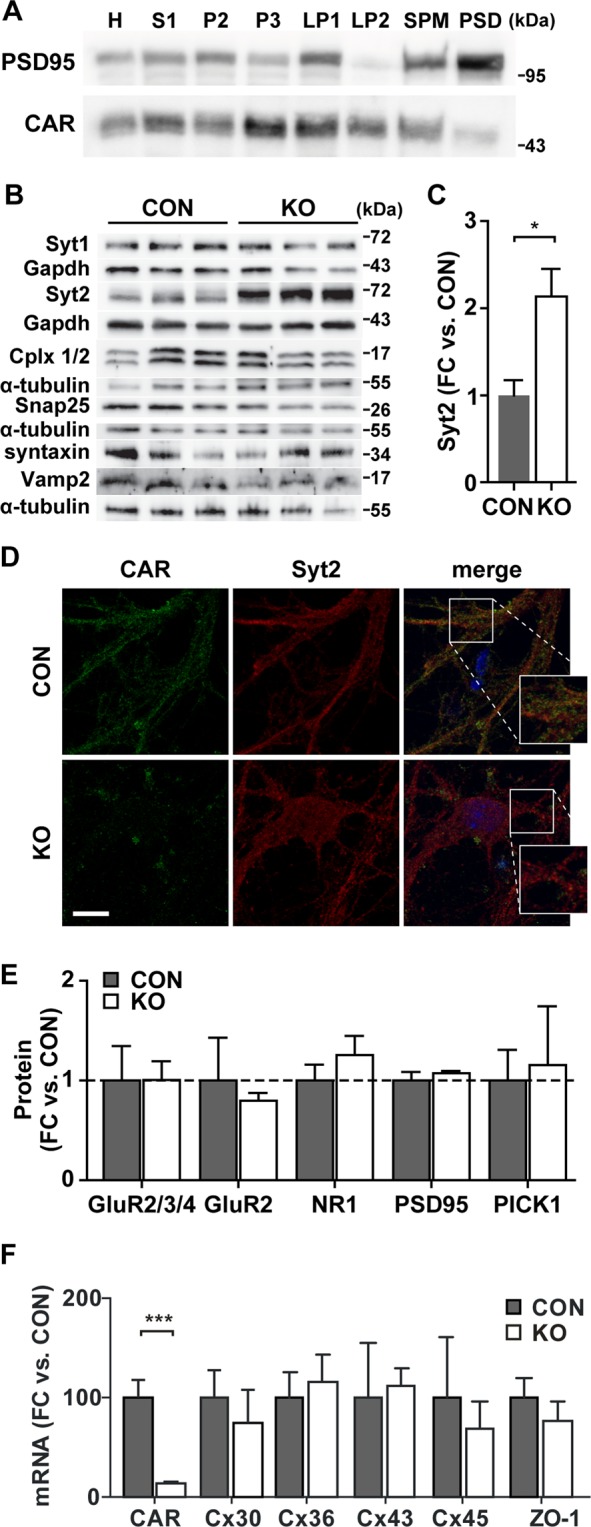
CAR deletion affects Syt2 expression. (**A**) Subcellular fractionation of P14 wild-type brain. Same amount of protein from each fraction was analyzed by Western blot for the presence of CAR (blots cropped from different gels). PSD95 was used as a control for proper fractionation and as a marker of the postsynapse. CAR is localized in all membrane fractions (H - homogenate, S1 - cell lysate, P2 - crude synaptosomal membrane, P3 - light membrane, LP1 - synaptosomal membrane, LP2 - synaptic vesicles, SPM - synaptic plasma membrane, PSD - postsynaptic density). (**B**) Presynaptic protein expression on 6-week-old hippocampi analyzed by Western blot (cropped from different gels). CAR KO animals show a specific increase in synaptotagmin 2 (Syt2) protein level without changes in further proteins involved in vesicle release. (**C**) Quantification of Syt2 expression from (**B**). Syt2 is increased two-fold in CAR KO hippocampi (*p < 0.05). (**D**) Immunofluorescence staining of wild-type and CAR KO hippocampal neurons. There is a partial co-localization of CAR (green) and Syt2 signal (red) in synapses (Scale bar, 25 µm). (**E**) Postsynaptic protein expression on 13-week-old hippocampi analyzed by Western blot. In the CAR KO mice none of the proteins tested was significantly changed in comparison to controls. (**F**) Quantitative RT-PCR. The gene expression levels of connexins and ZO-1 are unchanged in CAR-deficient brain from 3-weeks-old mice while CAR is downregulated (n = 4 per group; ***p < 0.001).

To refine our understanding of CAR at the synapse, we performed subcellular fractionation of 14-day-old mouse brains. We found CAR in almost all membrane fractions including the synaptosomal membrane fraction (LP1) and the fraction of synaptic vesicles (LP2). CAR was also present in the postsynaptic density (PSD), although in lower amounts (Fig. 5A), consistent with published localization data^41^. Thus, the presence of CAR at both sides of the synapse may suggest a role in neuronal communication that involves an effect on the cycling of synaptic vesicles as well as on postsynaptic effectors.

Towards understanding the molecular basis of the electrophysiological phenotype, we analyzed the expression of pre- and postsynaptic proteins in CAR KO and control hippocampi. Among the presynaptic proteins, there were no effects on the amount of Syt1, Cplx1 and Cplx2, Snap 25, syntaxin or Vamp2 protein, but we found a specific upregulation of Syt2 in CAR KO mice compared to controls (Fig. 5B). The Syt2 protein level in CAR KO hippocampi increased two-fold compared to control mice (Fig. 5C). Partial co-localization of CAR and Syt2 in cultured hippocampal neurons (Fig. 5D) suggests a similar subcellular distribution of these proteins. The colocalization was quantified using the thresholded Manders value (tM1 and tM2). CAR was set to channel 1 and Syt2 to channel 2 resulting in tM1 of 0.413 ± 0.036 (n = 36) and tM2 of 0.483 ± 0.034 (n = 36). The thresholded Manders values were calculated using the ImageJ plugin. Values for the colocalization of CAR and Syt2 were significantly below the colocalization of CAR and its binding partner β-catenin^42^ (tM1 0.626 ± 0.025, n = 25, p < 0.0001; tM2 0.652 ± 0.034, n = 25, p = 0.0013), but increased as compared to the negative control tubulin and synaptotagmin 2 (tM1 0.191 ± 0.024, n = 25; tM2 0.207 ± 0.024, n = 25). Since Syt2 is a calcium sensing protein interacting with core complex of vesicle docking^43,44^, we provide additional support that CAR deletion alters the presynaptic function of hippocampal neurons.

The interaction of CAR with the postsynaptic proteins PSD95 and PICK1^24^ prompted our analysis of CAR dependent postsynaptic protein expression. As markers of the postsynapse we analyzed the NR1-subunit of NMDAR as well as the GluR2-, GluR3-, and GluR4-subunits of AMPAR, since they interact with PSD95 and PICK1^26,28^. In addition these receptors play a role in synaptic transmission and LTP – both increased in our CAR KO mice^29,45^. Nevertheless, there were no changes in the protein levels of PSD95, PICK1, or the subunits of NMDAR and AMPAR in CAR KO compared to control mice (Fig. 5E).

In the heart-specific knockout of CAR, the impaired cell-cell communication is based on changes in function and expression of the gap junction forming proteins connexins^17,18^. Connexins (Cx) play an important role in astrocyte communication that provide feedback on neuronal function and the deletion of Cx30 and Cx43 in astrocytes negatively affects synaptic transmission and plasticity in CA1 pyramidal neurons^46^. To determine if the altered synaptic transmission in CAR deficient mice is based on changes in connexin expression, we measured mRNA levels of Cx30, Cx36, Cx43 and Cx45 in brains from three-week-old CAR KO and control mice. We also included ZO-1 into our analysis, as it links CAR to connexins. Their expression in CAR KO brain versus controls was unchanged – while CAR mRNA levels were reduced to 10% of wild-type levels (Fig. 5F). This indicates that CAR affects cellular communication in the brain through the interaction with other proteins than in the heart.

### Molecular changes of CAR KO mice involve sensory perception and G-protein receptor signaling

**Figure 6:**
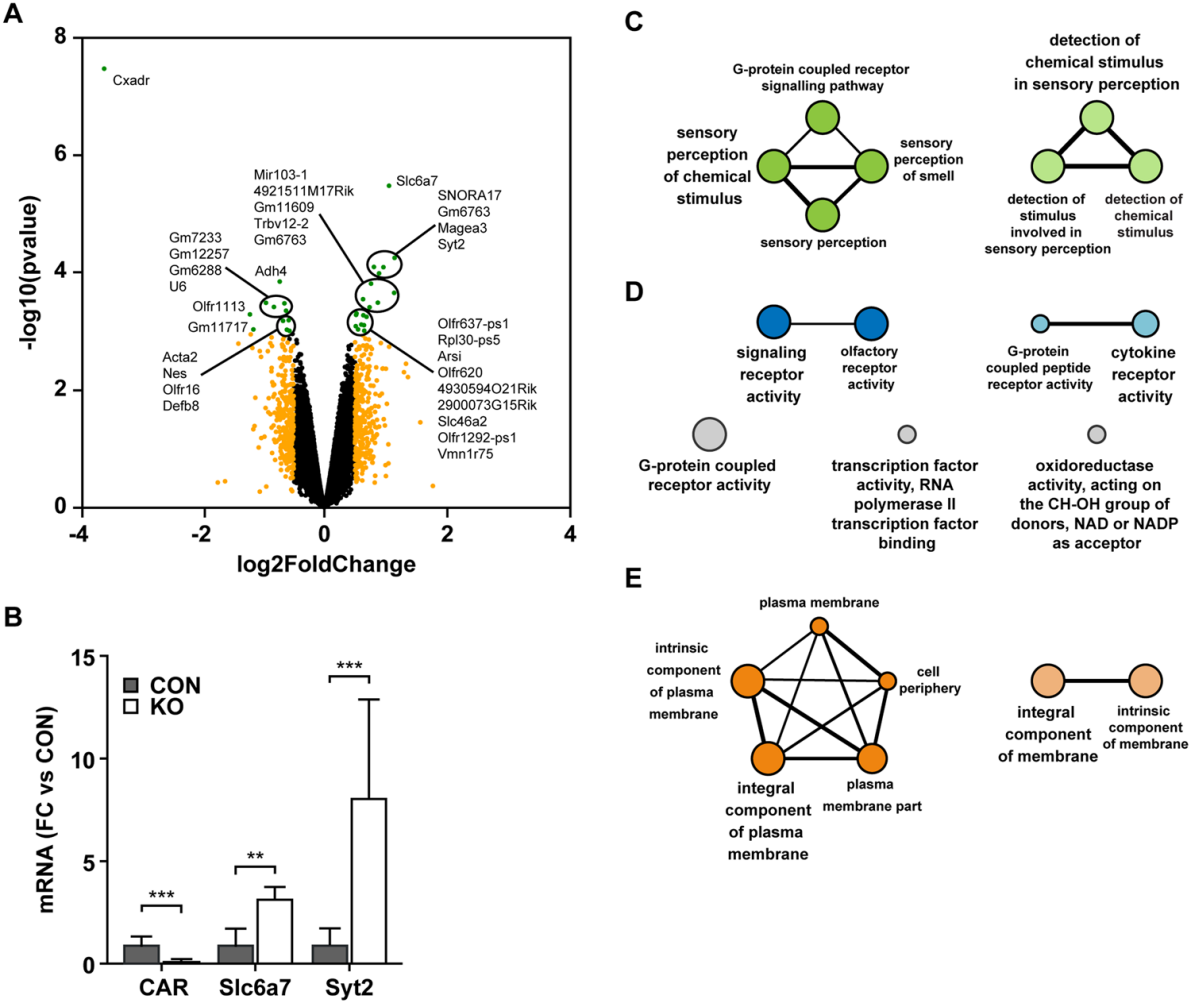
Gene expression changes associated with loss of CAR. (**A**) Volcano plot of the log2-fold change of gene expression levels (X-axis) against the –log10 p-value (Y-axis) comparing 10 day old hippocampi from CAR KO and control mice. Genes with absolute log2 fold changes >0.5 are shown in orange. Significantly regulated genes (p-values < 0.001 and absolute log2 fold changes >0.5) are shown in green (left downregulated; right upregulated). (**B**) qRT-PCR. At postnatal day 7 (P7) *Slc6a7* and *Syt2* mRNA-levels are significantly upregulated in KO hippocampus compared to controls (CON) (FC, fold change). (**C**) Gene ontology analysis of significantly regulated genes (p-values < 0.001 and absolute log2-fold changes >0.4). Cytoscape ClueGO was used to perform clustering analysis for (**C**) biological processes, (**D**) molecular function and (**E**) cellular compartment. The grouping revealed enrichment for sensory perception and G-protein-coupled signaling as well as for plasma membrane.

To expand our molecular analysis of the CAR KO strain, we performed an exon chip analysis on hippocampi of 10-day-old KO and control animals. We visualized CAR dependent gene expression as a volcano plot (Fig. 6A). We confirmed the upregulation of Syt2 and Slc6a7 mRNA (Fig. 6B). Additionally, the expression of several olfactory receptors was different between CAR KO mice versus controls (Supplementary Table 1). Using the Cytoscape plugin GlueGO, we clustered the significantly regulated genes (log2 fold change cutoff 0.4 and p value < 0.05) into functional networks. Grouping for biological processes and molecular function revealed that the genes are involved in sensory perception and G-protein receptor signaling (Fig. 6C,D). As expected, the major cellular localization of regulated proteins is the plasma membrane (Fig. 6E). Together the results indicate that in the CAR KO animals, molecular changes localize to both pre- and postsynaptic sites, confirming the electrophysiology data.

### CAR interacts with proteins of the exocytosis machinery

To link CAR to the vesicle transport and postsynaptic signal transduction, we performed a yeast-two-hybrid screen (Y2H) and a BioID-assay to combine interaction and localization data.

**Figure 7:**
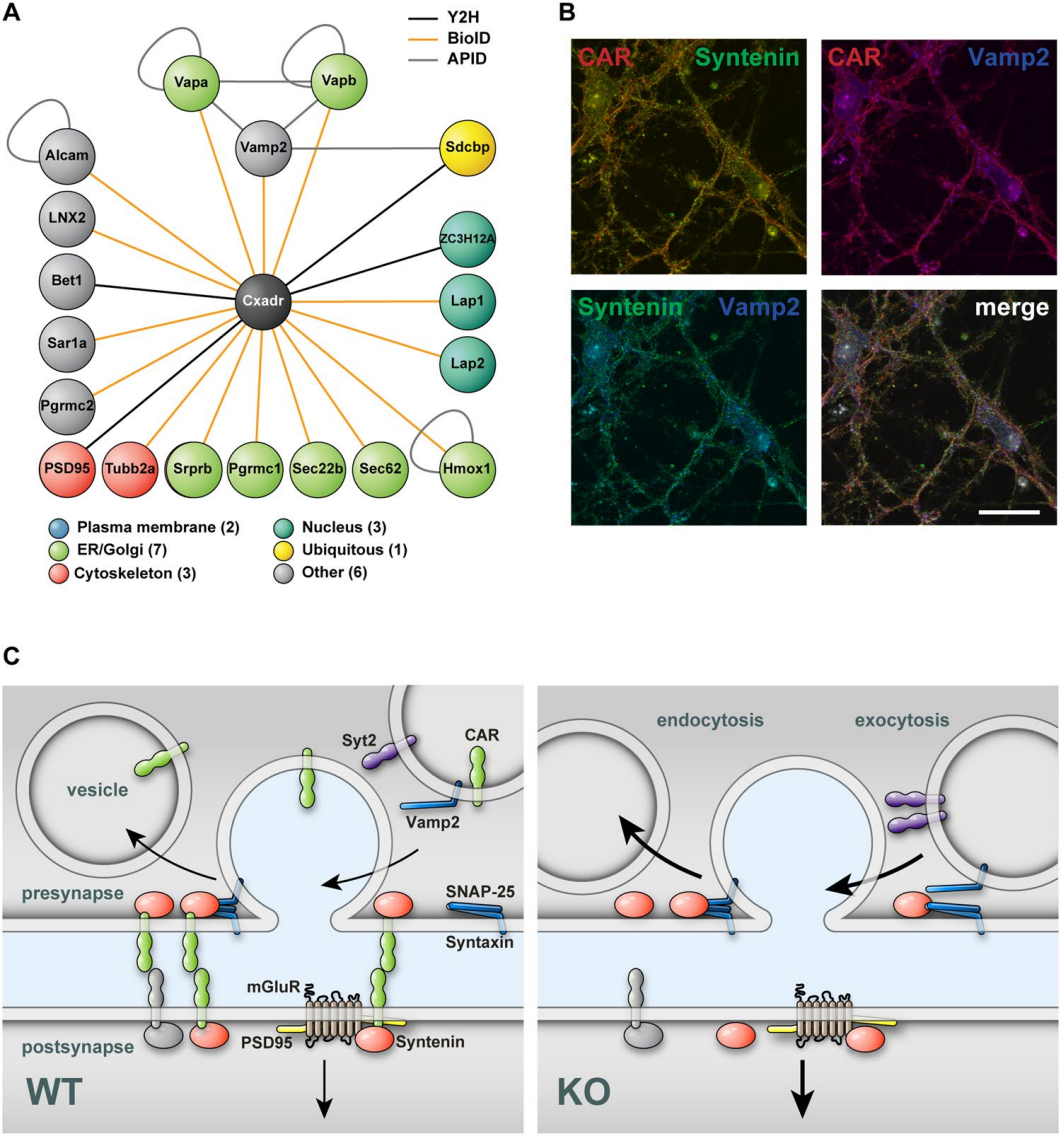
CAR integrates synaptic functions. (**A**) Cytoscape APID2NET analysis of BioID- and Y2H-experiments. CAR (shown in black) interacts with proteins (node) of different compartments as identified by BioID-assay (PEP < 0.01, orange line color) or Y2H screen (hits > 4 SDIV repeats, black line color). Grey color indicates Cytoscape plugin APID database records. Cellular compartment was attributed using the COMPARTMENTS software. Proteins with more than one compartment were named ‘ubiquitous’. Proteins localized to compartments other than plasma membrane, ER, Golgi, cytoskeleton or nucleus were named ‘other’. The analysis revealed new synaptic interaction partners of CAR, such as Vamp2/VapA/VapB or Sdcbp (syntenin). (**B**) Immunofluorescence of isolated wild-type hippocampal neurons (DIV 13) indicates partial colocalization of CAR (red) with syntenin (green) and Vamp2 (blue) as well as syntenin with Vamp2 (Scale bar, 25 µm). (**C**) Model of CAR function on the presynapse where sequestration of Syntenin (red) reduces the likelihood of vesicle release and the speed of endocytosis. The compartmentalization of the synapse through CAR (green) restricts mobility of postsynaptic proteins such as glutamate receptors (mGluR) reducing synaptic transmission. CAR can homo- or heterodimerize depending on the protein composition of the postsynaptic side (green green vs. green/grey).

For the Y2H screen we used the human CAR cytoplasmic tail fused to a LexA DNA-binding domain as bait construct and a human brain prey library in an automated Y2H-setup^47^. Interactions were weighted according to the number of reproducibility of diploid yeast clones growth (repeats) on SDIV selection medium that contains the corresponding protein-protein interaction between bait and prey with 4 being the highest repeat and 1 the lowest. We identified PSD95 and the ligand of numb-protein X 2 (LNX2) as CAR interacting proteins (SDIV repeat = 4), confirming prior findings^23,24^. Further proteins with a SDIV repeat of 4 are the syndecan-binding protein (SDCBP, also known as syntenin) and the zinc finger CCCH-domain containing 12 A protein (ZC3H12A), which have not been identified as CAR interaction proteins before (Fig. 7A, Supplementary Table 2). PSD95, LNX2 and syntenin (SDCBP) contain at least one PDZ-domain pointing towards an interaction with the C-terminal PDZ-domain binding motif of CAR.

For the BioID-Assay we used a full length CAR construct with an in-frame insertion of the BirA* biotin ligase between the transmembrane region and the cytoplasmic tail of CAR. Differentiated neuron-like PC12 cells stably expressing the CAR-BirA* fusion protein were incubated with biotin after the formation of dendrites. This lead to the biotinylation of closely localized and interacting proteins through the BirA* biotin ligase. These proteins were then purified with streptavidin beads and identified by mass spectrometry. Using differentiated, untransfected PC12 cells incubated with biotin as a control we excluded unspecific biotinylation by endogenous biotin ligases. With this technique we identified several novel proteins located proximal to CAR (Supplementary Table 3).

To classify the proteins identified in our screens, we performed bioinformatics analysis with Cytoscape 2.8.2 and the APID2NET plugin. We focused on proteins with a SDIV repeat greater than 2 (Y2H screen) and a PEP score less than 0.01 (BioID-assay), respectively (Supplementary Tables 2 and 3). We assigned a subcellular localization of the candidate proteins using the COMPARTMENTS software.

Many of the identified proteins localized to the ER/Golgi network, plasma membrane and the cytoskeleton (Fig. 7A). Interestingly, syntenin (Sdcbp), identified in our Y2H screen (black line color), directly interacts with the vesicle associated membrane protein Vamp2 (also known as synaptobrevin 2), identified by the BioID-assay (orange line color) which in turn interacts with the vesicle-associated membrane protein-associated protein A (Vapa) and B (Vapb). Nearest neighbor analysis using the same plugin reveals that synaptobrevin/VAMP2, Vapa, and Vapb are embedded in a network of proteins relevant for synaptic vesicle exocytosis and that syntenin is linked to this network not only by direct interaction with Vamp2 but also via the shared interaction partners syntaxin 1 A (STX1A) and synaptosomal-associated protein 25, Snap 25 (SNP25). Building on the syntenin and Vamp2 interaction data, we studied their colocalization with CAR using immunofluorescence staining of cultured hippocampal neurons. Indeed, we documented partial colocalization of CAR and syntenin as well as CAR and VAMP2 (Fig. 7B), based on the quantification with similar thresholded Manders values. For CAR (channel 1) and syntenin (channel 2) we measured tM1 0.521 ± 0.040 (n = 48) and tM2 0.529 ± 0.046 (n = 48). For CAR (channel 1) and VAMP (channel 2) we found tM1 0.559 ± 0.053 and tM2 0.564 ± 0.06 (n = 28).

Taken together, we confirmed the interaction of CAR with postsynaptic proteins and uncovered new interactions of CAR with proteins of the presynapse – in particular those related to vesicle exocytosis.

## Discussion

CAR is crucial for normal embryonic development and electrical conductance in the adult heart. Here we describe a novel role of CAR in neuronal cell-cell communication using a conditional knockout approach to exclusively study the role of CAR in the postnatal brain. Firstly, neuronal deletion of CAR at early postnatal stages enhances synaptic transmission and LTP as well as the probability of synaptic vesicle exocytosis and the speed of endocytosis. Secondly, our data on synaptic protein expression and CAR distribution in synaptic membrane fractions extend previous work that attributed a primary postsynaptic function of CAR^24^. Thirdly, our interaction data and colocalization of CAR with syntenin, Vamp2, Vapa, and Vapb link CAR to proteins involved in neurotransmitter release. Additionally, in the hippocampus of CAR KO animals we find a specific upregulation of Syt2, a protein crucial for calcium induced exocytosis and, possibly, also for endocytic recycling^48–50^. Thus, our data extend the role of CAR in the adult brain with a novel role at the presynapse *in vivo*.

Cell adhesion proteins are important for neurite outgrowth and neuronal cell-cell contact formation during brain development^3,51,52^. Dendrite arborization and morphology of synapses, as well as the ratio of inhibitory to excitatory neurons are unchanged in CAR KO brains upon CAR deletion from the early postnatal stage. This lack of morphological change in the adult animal parallels our previous findings in the inducible heart specific CAR KO mice which display proper cardiac cell contacts^18^. In both the brain specific CAR KO and the inducible heart specific CAR KO, CAR is deleted after the initial formation of cell-cell contacts. We suggest that early postnatal elimination of CAR does not affect preexisting cell adhesions in either tissue. This is in line with findings of conditional deletion of the IgSF proteins L1 and NCAM which have normal brain morphology in contrast to the conventional germline-induced L1 or NCAM KO mouse strains^52–54^. Unlike other IgSF members, CAR is largely dispensable in the developing brain, as animals deficient for CAR in all tissues but the heart survive without an obvious phenotype^55^. Thus, the enhanced synaptic transmission most likely results from a primary function of CAR in the mature neuron and is not secondary to developmental defects. These CAR-mediated changes in synaptic function does not result in changes in the behavior tests, in contrast, when CAR is knocked out in early embryonic stages the deficiency leads to sex specific behavioral changes restricted to females only^30^.

We found the presynaptic proteins synaptobrevin/VAMP2, Vapa and Vapb localized in the proximity of CAR and identified syntenin as a so far unknown interaction partner of CAR. Syntenin is a scaffold protein, which interacts with cell adhesion molecules and synaptic proteins. It contributes to the molecular organization of the active zone by interacting with CAST1, a protein of the cytomatrix in the active zone^56^. Additionally, the interaction of syntenin with ephrin-B1 and ephrin-B2 contributes to the development of the presynapse^57^. Therefore, we suggest that syntenin localizes CAR at the active zone and mediates its effect on vesicle release. Indeed, a similar increase in neurotransmitter release as documented in the CAR knockout has been observed in animals deficient in the syntenin binding protein CAST^58^.

Furthermore, syntenin, as well as the CAR binding protein Pick1 interact with postsynaptic proteins such as glutamate receptors, participating in their targeted distribution at the synapse^59^. Loss of CAR could lead to an imbalance in Pick1 and syntenin function at the postsynaptic side with altered compartmentalization of glutamate receptors - the receptors that facilitates LTP^60^.

CAR is one of the few IgSF proteins involved in vesicle exo- and endocytosis – among them CHL1, NCAM and alpha-neurexin. Interestingly their molecular function is quite diverse: CHL1 affects the interaction of chaperones with SNARE proteins and thus transmitter release. NCAM is involved in vesicle mobilization and cycling through the regulation of the myosin light chain kinase signaling^10,61^. Alpha-neurexin localizes calcium channels to sites of vesicle release^62^. Since CAR as well as neurexin and SynCAM1 interact with the scaffold protein syntenin, CAR might follow a similar mode of action^56,63,64^. SynCAM promotes synapse formation^8,64^ - unlike CAR, where the number of synapses is unchanged in KO mice. Therefore, we suggest that CAR predominantly acts on the presynaptic side at the level of the individual synapse, based on our findings that in CAR KO mice, synapse number, vesicle density and all other morphological parameters except for the vesicle number near the active zone was normal. This is supported by co-localization of CAR with several proteins crucial for vesicle exo- and endocytosis and a specific upregulation of Syt2 expression in CAR KO neurons. Syt2 is a structural and functional homologue of Syt1 and both proteins are calcium sensors, which trigger calcium-dependent vesicle exocytosis^44,65,66^. They help to position vesicles adjacent to calcium channels, which affects priming and stabilization of the primed pool^67,68^. Syt1 is primarily expressed in the forebrain, while Syt2 expression is mainly restricted to the caudal regions of the brain and largely suppressed in hippocampus and cortex^69,70^. As direct effects of CAR on the regulation of gene expression have so far not been described, increased Syt2 levels are likely secondary effects reflecting altered vesicle mobility or priming and might contribute to improved transmitter release and thus synaptic strength.

Our data suggest a novel predominantly presynaptic function of CAR. Feedback signaling from the post- to the presynapse has previously been reported for the cell adhesion proteins neurexin and cadherin^71,72^ and synCAM provides an example for transsynaptic homodimerization^64^. Our BioID approach extends the synaptic proteome and is consistent with a role of CAR homo- or heterodimers as additional links across the synaptic cleft.

In summary, we show that CAR is a novel inhibitor of synaptic transmission that acts primarily on the presynaptic side with functional consequences for excitatory synaptic transmission and vesicle cycling in postnatal CAR knockout mice.

## Methods

### Animals

CAR^lox/lox^ mice have been previously described^73^ and were mated with CamKIIα-Cre transgenics to generate a brain specific CAR KO model.

All experiments involving animals were performed in accordance with relevant guidelines and regulations (Care and Use of Laboratory Animals of the German Animal Welfare Act) after approval by the local authorities (Landesamt für Gesundheit und Soziales - LaGeSo Berlin).

Mice in all groups were housed in the animal facility under 12:12 light/dark cycles and had free access to water and pelleted food twenty-four hours a day. Western blots, cell- or slice preparations were covered by license T403/09 and the behavior tests by license G0073/10.

The analysis of postnatal adaptation was performed in the first months of age and all tissue culture work was done at ~14 days *in vitro*). For behavioral analysis and field recordings with matching gene expression, we used adult mice. Analysis of function (mEPSC), morphology, and gene expression in postnatal and adult animals is described below and in the online supplement.

### Gene expression analysis

Total RNA of the mouse hippocampus was isolated with TRIZOL (Invitrogen Corp.), purified using the RNeasy Micro Kit (QIAGEN), and used for cDNA synthesis and qRT-PCR as described previously (Lisewski *et al*., 2008). The Affymetrix Exon Mouse Chips 1.0 ST were used for the gene expression analysis in the hippocampus of the 10-day-old mice following the manufacturer’s instructions.

For protein preparation, SDS PAGE, and Western blot with ECL-detection we followed our published protocols^18^. Additional details and information on the qRT-PCR probes and antibodies is available in the online supplement.

### Morphology

Brains from 7-day-old mice were fixed in 4% formaldehyde overnight, washed with PBS, and kept in PBS with 30% sucrose at 4 °C. The brains were cut in 40 µm floating sections for immunohistochemistry and processed as described in the online supplement.

For immunofluorescence, cells were fixed in 4% formaldehyde for 15 min, permeabilized and stained as outlined in the online supplement. For electron microscopy, isolated hippocampal neurons were fixed in 3% formaldehyde and 2.5% glutharaldehyde in 0.1 M cacodylate-buffer, dehydrated in a graded ethanol series, embedded in Epon, cut in 70 nm sections and contrasted with uranyl- and lead-citrate. Pictures were taken with a Zeiss 910 electron-microscope and a CCD camera (Proscan) and analyzed using the iTEM software (Olympus Soft Imaging Solutions).

### BioID-assay

The BioID assay was performed based on the description of Roux *et al*.^74^. The CAR-BirA* construct was stable transfected into PC12 cells using zeocin as selection antibiotic. Details on the DNA constructs and the assay is available online. In brief, stable BioID clones were differentiated for 3 days with 50 ng/ml neural growth factor (Sigma-Aldrich) prior to incubation with 50 µM biotin for 3 days. Biotinlylated proteins were enriched using Streptavidin-Dynabeads and identified by Massspectrometry.

### Yeast-two-hybrid screen

The automated Yeast-two-hybrid screen was performed as described previously^47^. The pBTM116CAR-tail plasmid was tested for autoactivity prior to screening the human brain prey-library.

### Live cell imaging and electrophysiology

Hippocampal neurons were isolated following a modified protocol from Bekkers and Stevens^75^, transfected at 7–8 days *in vitro* and imaged at 14–15 div after electric field stimulation with 200 action potentials (at 5 Hz, 100 mA) and stimulation-induced pHluorin responses were recorded to characterize exocytosis as detailed in the online supplement. We used the decay function of one trace to compensate for the endocytic component of the same trace^36^. Release rates were obtained from the ratio of an average trace and the exponential fit on its decay phase, calculated in Fourier space, using the MATLAB functions “fast-Fourier transformation” and “inverse fast-Fourier transformation”. Values were plotted as cumulative release over time.

To record extracellular neuronal field responses, hippocampal slices of 3-month-old animals were prepared as previously described^76^. Field and mEPSC recodings are described in detail online.

### Behavior and learning

Barnes Maze analysis was performed as described in the online supplement with mice trained for four days to find the hidden box positioned under the target hole. For context-fear-conditioning the conditioned stimulus (CS, tone, 60 db, 10 kHz, 50 ms rise time) was applied twice for 15 s together with the unconditioned stimulus (US, electrical foot shock, 0.7 mA). Mice were reanalyzed on the next day first in the same box, then after 2 h break in a new box with different shape, light and odor. After three minutes the tone was applied for 3 min. The movement of mice during the conditioning, context and cue analysis was observed via the camera of the context-fear-conditioning setup and the percentage of freezing was calculated by the Med-Software (Med Associates). The Open Field test was carried out in the native cage environment. Statistical analysis was performed using unpaired-t test by GraphPad. Additional details are available online.

### Statistical analysis

For statistical analysis, we used GraphPad Prism 5.0. Results are expressed as means ± SEM. Statistical significance between groups was determined using the Mann Whitney U test for electrophysiology data. Expression values were compared using an unpaired two-tailed t test to assess differences between two groups. For comparison of more than one group the One-Way-ANOVA and for more than one condition the Two-Way-ANOVA was used. The significance level was chosen as p = 0.05.

All data generated or analyzed during this study are included in this published article and the Supplementary Information files.

## Supplementary information

Supplement
