# Supplementary material for: The cell adhesion protein CAR is a negative regulator of synaptic transmission
Source: Sci Rep. 2019 May 1;9:6768. doi: 10.1038/s41598-019-43150-5 (PMC6494904; doi:10.1038/s41598-019-43150-5)
Supplement: Supplementary file 1 — Supplement [file 41598_2019_43150_MOESM1_ESM.docx]

# The cell adhesion protein CAR is a negative regulator of synaptic transmission

Uta Wrackmeyer, Joanna Kaldrack, René Jüttner, Ulrike Pannasch, Niclas Gimber, Fabian Freiberg, Bettina Purfürst, Dagmar Kainmueller, Dietmar Schmitz, Volker Haucke, Fritz G. Rathjen and Michael Gotthardt

# Supplementary Methods

## Animals

CAR^lox/lox^ mice have been previously described (Shi et al., 2009). Animals with a floxed CAR exon 1, which contains the ATG, were mated with CamKIIα-Cre transgenics to generate a brain specific CAR KO model. CamKIIα-Cre transgenic mice were obtained from EMMA (www.emmanet.org). All experiments involving animals were performed in accordance with relevant guidelines and regulations after approval by local authorities (LaGeSo Berlin). Data from both male and female animals were combined for data analysis.

## DNA constructs

For the generation of the CAR-BirA* fusion protein, the appropriate CAR coding regions were amplified from mouse brain cDNA. BirA* was amplified from the plasmid pcDNA3.1 mycBioID (Addgene). DNA fragments were assembled by PCR-based gene assembly fusing the BirA biotin ligase gene between the transmembrane CAR region and the cytoplasmic tail. The CAR-BirA* construct was then cloned into the pcDNA3.1/Zeo+ expression vector (Invitrogen). For the Y2H screen DNA coding for the human CAR tail was amplified from human Hela cDNA and cloned in frame into the modified pBTM116 bait-vector (gift from Erich Wanker, MDC) to generate the LexA-CARtail fusion protein. For the synaptopHluorin imaging we used the Syp-pHluorin (rat) from Leon Lagnado ^1^.

## Antibodies

CAR antibody used for immunofluorescence staining is published ^2^. The following antibodies are commercially available: CAR, Snapin (Santa Cruz); β-Tubulin (Sigma); GAPDH (Chemicon); NR1, Synaptophysin, Syt1, Syt2, Syntenin, Vamp2, Cplx 1/2, vGlut, GluR2/3/4 (Synaptic Systems); MAP2 (Abcam); GAD 65/67 (Millipore), PSD95 (Abcam), PICK1 (UC Davis).

## qRT-PCR probes

CAR and 18s-RNA primers are published ^3^. The following probes are commercially available at Applied Biosystems: Cx30, Cx36; Cx43, Cx45, Syt2, ZO-1.

## Isolation of hippocampal neurons

Hippocampal neurons were isolated following a modified protocol from Bekkers and Stevens^4^. In short: Brains of two-day-old newborn mice were isolated and hippocampi were dissected in cold HBSS buffer and incubated in 1 ml Enzyme-Solution (2 mg L-cysteine (Merck); 50 ml DMEM (Invitrogen); 1 mM CaCl_2_; 0.5 mM EDTA and 25 U/ml Papain (cellcystems) for 1h at 900 rpm, 37°C. The Enzyme-Solution was exchanged for 1 ml Stop-Solution (2.5 mg/ ml Albumin (Sigma); 2.5 mg/ ml Trypsin Inhibitor (Sigma) in Dissociation-Solution) and incubated for 5 min. Hippocampi were mechanically dissected with Dissociation-Solution (DMEM; 5% FCS; 100 U/ml Pen; 0.1 mg/ml Strep), and centrifuged at 100x g for 10 min. The cell pellet was resuspended in Neuron-Medium (Neurobasal A (Invitrogen); 2% B27 (Invitrogen); 1% Glutamax (Invitrogen); 100 U/ml Pen; 0.1 mg/ml Strep) and plated on Poly-D-Lysin/Collagen (Sigma) coated glass cover slips or dishes. After two days FUDR (8.1 mM 5-fluoro-2‘-desoxyuridine, 20.4 mM uridine (Sigma); 12.5 ml DMEM) was added. Medium was changed every three days.

## Quantitative Real time PCR

Total RNA of the mouse hippocampus or cortex was isolated with TRIZOL (Invitrogen Corp.), purified using the RNeasy Micro Kit (QIAGEN), and used for cDNA synthesis and qRT-PCR as described previously (Lisewski et al., 2008).

## Microarray hybridization and data analysis

The Affymetrix Exon Mouse Chips 1.0 ST were used for the gene expression analysis in the hippocampus of the 10-day-old mice. 1 µg total-RNA was processed with the RiboMinus Transcriptome Isolation Kit (Human/Mouse), RiboMinus Concentration Module, WT Sense Target Labeling and Control Reagents Kit and the GeneChip Hybridization, Wash and Stain Kit. The chips were scanned with the Affymetrix GeneChip Scanner 3000. The data was analyzed with the Affymetrix GeneChip Operating Software and Altanalyze. Volcano plot was generated from all detected genes by plotting the p value of the t-Student test against log2 fold change of gene expression.

## SDS-Page and Western blot

For protein preparation, SDS PAGE, and Western blot with ECL-detection we followed our published protocols ^3^. We used primary antibodies directed against CAR (rabbit polyclonal, Santa Cruz) and γ-tubulin (mouse monoclonal, Sigma), Syt2 (rabbit polyclonal, Synaptic Systems), PSD95 (mouse monoclonal, Upstate), GAD65/67 (rabbit, Millipore), vGlut1 (mouse monoclonal, Synaptic Systems), syntaxin (mouse monoclonal, Millipore), synapsin (mouse monoclonal, Millipore), munc18 (rabbit, Synaptic Systems) and GAPDH (mouse monoclonal, Affinity BioReagents) according to the manufacturer’s instructions. Quantification was performed with the Aida Imager Analyzer v4.19 software.

## Immunohistochemistry

Brains from 7-day-old mice were fixed in 4% formaldehyde overnight, washed with PBS, and kept in PBS with 30% sucrose at 4°C. The brains were cut in 40 µm floating sections using the Leica SM2000R Sliding Microtome and stored in CPS (0.1 M Na-Phosphate-Buffer, pH 7.4; 25% glycerol; 25% ethylene glycol) at 4°C. Sections were blocked for 1h in 2% BSA, 2% donkey serum in PBS and incubated with anti-CAR antibody (rabbit polyclonal) overnight at 4°C. Endogenous peroxidases were inactivated by a 15 min treatment with 3% H_2_O_2_ in PBS. Slices were washed in PBS, incubated with the secondary biotin-conjugated donkey anti-rabbit antibody (Jackson ImmunoResearch Laboratories), and developed with the ABC Elite reagent (Vector Laboratories, Burlingame, CA) and DAB (Dako).

## Immunofluorescence staining

Cells were fixed in 4% formaldehyde for 15 min, washed twice with PBS and permeabilized with 0.3% Triton X-100 in PBS for 15 min. After 1 hour blocking in 2% BSA, 2% donkey serum in PBS cells were incubated with the primary antibody at 4°C overnight, washed with PBS and incubated with secondary-fluorescence antibody (goat anti-rabbit Alexa 488, Chemicon) at room temperature for 2 h. After washing the cells were blocked and incubated with the 2^nd^ primary antibody followed by washing and the 2^nd^ secondary biotin-conjugated antibody (Jackson Immuno Research) and streptavidin-568 (Invitrogen) after washing. Primary antibodies used: anti-Syt2 antibody (rabbit) and anti-CAR.

## Quantitative colocalization analysis

Quantitative colocalization analysis was performed using the ImageJ-1.51s software (National Institutes of Health, Bethesda, MD) with the “Coloc2” plugin, which allows calculation of the overlapping pixels intensity between the two channels representing the degree of colocalization. The thresholded Mander’s values and the Costes p-values were recorded - data with Costes p-value of 1.0 were accepted only. The fluorescence of CAR was detected in channel 1 whereas Syt2, VAMP or syntenin was detected in channel 2.

## Electron microscopy

Isolated hippocampal neurons were plated on 35 mm Lumox dishes (Greiner) and fixed in 3% formaldehyde and 2.5% glutharaldehyde in 0.1 M cacodylate-buffer for 4h. Cells were washed with cacodylate-buffer, treated with 1% osmiumtetroxide pH 2.5 for 2 h, and dehydrated in a graded ethanol series. Cells were embedded in Epon, cut in 70 nm sections and contrasted with uranyl- and lead-citrate. Pictures were taken with a Zeiss 910 electron-microscope and a CCD camera (Proscan) and analyzed using the iTEM software (Olympus Soft Imaging Solutions).

For analysis of synapse density in slices, wild type and CAR KO mice were perfused with freshly prepared 4 % formaldehyde in 0.1 M phosphate buffer. Pieces of the hippocampus were postfixed with 2% formaldehyde/2.5% glutaraldehyde in phosphate buffer for 2 days, treated with 1 % osmiumtetroxide, dehydrated in a graded ethanol series and propylene oxide, and embedded in Poly/BedR 812 (Polysciences, Inc.). Ultrathin sections (70 nm) from the CA1 region of the hippocampus were contrasted with uranyl acetate and lead citrate and analyzed in a Zeiss 910 electron microscope, equipped with a Quemesa camera and the iTEM software (EMSIS GmbH, Münster)). Ten photos per section were taken randomly at a magnification of 8000x, and the number of synapses per area was counted. Synapses were identified when apposed membranes occurred closely together, with an electron-dense paramembranous specialization on one or both sides of the intervening cleft, and the presence of three or more vesicles proximal to apposed membranes in the presumptive presynaptic profile. 80 view fields were counted from each mouse (one wild-type and two KO mice), respectively. One view field represents 32 µm^2^.

## Automated image analysis

Synapses were counted manually or using automated image analysis (convolutional neural network with U-Net architecture ^5^). Training data was derived from manual annotations of synapse center point coordinates in 20 view fields of a single animal, as follows: The Euclidean distance transform of synapse coordinates was computed per view field, and the resulting distance map per view field was filtered with a tanh() function scaled by a heuristically determined average synapse diameter ^6^. The U-Net was then trained to directly predict filtered distance transforms, from which center point locations were obtained in turn by detecting local minima below a threshold p, together with non-minimum suppression to avoid duplicate detections. Threshold p as well as the number of training epochs were determined automatically via model validation on the manual synapse counts from the 60 remaining view fields acquired for the training animal. The above procedure was run twice: once with a wild type mouse as the training animal, and once with a KO mouse as the training animal.

## BioID-assay

The BioID assay was performed based on the description of Roux et al. ^7^. The CAR-BirA* construct was stable transfected into PC12 cells using zeocin as selection antibiotic. Stable clones were expanded on two collagen coated (100 µg/ml) 15 cm dishes as well as normal PC12 cells (negative control) and differentiated for 3 days with 50 ng/ml neural growth factor (Sigma-Aldrich) prior to incubation with 50 µM biotin for 3 days. Cells were harvested with lysis buffer (50 mM Tris, pH 7.4, 500 mM NaCl, 0.4% SDS, 5 mM EDTA, 1 mM DTT, and 1x Complete protease inhibitor [Roche]) followed by sonication. After addition of Triton X-100 to 2% final concentration cells were further sonicated. An equal volume of 4°C 50 mM Tris (pH 7.4) was added followed by additional sonication (subsequent steps at 4°C) and centrifugation at 16.000 rcf. Supernatant was incubated with 600 μl Dynabeads (MyOne Steptavadin C1; Invitrogen) overnight. Beads were washed twice for 8 min at 25°C (all subsequent steps at 25°C) in 1 ml wash buffer 1 (2% SDS in dH_2_O), once with wash buffer 2 (0.1% deoxycholate, 1% Triton X-100, 500 mM NaCl, 1 mM EDTA, and 50 mM Hepes, pH 7.5), once with wash buffer 3 (250 mM LiCl, 0.5% NP-40, 0.5% deoxycholate, 1 mM EDTA, and 10 mM Tris, pH 8.1) and twice with wash buffer 4 (50 mM Tris, pH 7.4, and 50 mM NaCl). Beads were incubated 15 min 95°C with Lämmli-buffer including 3 mM biotin. Supernatant was loaded on a SDS-page, lanes were cut out and analysed by Massspectrometry.

## Yeast-two-hybrid screen

The automated Yeast-two-hybrid screen was performed as described previously ^8^. The pBTM116CAR-tail plasmid was tested for autoactivity prior to screening the human brain prey-library.

## Live cell imaging of hippocampal neurons using synaptopHluorin

Primary hippocampal neuron cultures were transfected at 7–8 days in vitro by a modified calcium phosphate transfection protocol (Promega). Neurons were imaged at 14-15 div.

Images were acquired using an inverted fluorescence microscope (Eclipse Ti, Nikon), controlled by MicroManager 4.11, equipped with a 40x oil-immersion objective (NA = 1.30, Nikon), a sCMOS camera (Neo, Andor) and a 200 Watt mercury lamp (Lumen 200, Prior). Images were acquired at 0.5 Hz with an EGFP filter set (F36-526, AHF Analysentechnik). Imaging was performed at room temperature in physiological imaging buffer (170 mM NaCl, 3.5 mM KCl, 5 mM NaHCO3, 5 mM glucose, 1.2 mM Na2SO4, 1.2 mM MgCl2, 1.3 mM CaCl2, 0.4 mM KH2PO4, 20 mM TES (2-[(2-Hydroxy-1,1-bis(hydroxymethyl)ethyl)amino]ethanesulfonic acid), pH 7.4). 50 µM APV (DL-2-Amino-5-phosphonopentanoic acid, Sigma-Aldrich) and 10 µM CNQX (6-Cyano-7-nitroquinoxaline-2,3-dione, Sigma-Aldrich) were added to the imaging buffer to silence spontaneous neurotransmission. Neurons were stimulated by electric field stimulation with 200 action potentials (at 5 Hz, 100 mA) in a stimulation chamber (RC-47FSLP, Warner Instruments) and stimulation-induced pHluorin responses were recorded.

The pHluorin intensity signal over time was measured from regions of responding synapses.

Deconvolution of pHluorin traces: In order to further characterize exocytosis by pHluorin experiments, components from endocytosis and reacidification during the stimulation were compensated by deconvolution. All intensity traces of an individual experiment were averaged. The decay phase of average pHluorin intensity traces, after the end of stimulation, was fit with an exponential decay function (equation 1).

*Exponential Decay:* $I\left( t \right)=bg+a{\cdot e}^{-\tau\cdot t}$ (1)

I(t) : Intensity at time t; bg: background signal; a: intensity at time 0; τ: lifetime

The fitted results were used as a template to deconvolve the intensity trace of the same stimulus. In contrast to earlier studies which used the decay function of a calibration stimulus for the deconvolution of a second pulse, we used the decay function of one trace to compensate for the endocytic component of the same trace ^9^. Release rates were obtained from the ratio of an average trace and the exponential fit on its decay phase, calculated in Fourier space, using the MATLAB functions “fast-Fourier transformation” and “inverse fast-Fourier transformation”. Values were plotted as cumulative release over time.

## Electrophysiology

### Field recordings

To record extracellular neuronal field responses, hippocampal slices of 3-month-old animals were prepared as previously described ^10^. Mice were anesthetized with isoflurane and the brains were removed. Tissue blocks containing the subicular area and hippocampus were mounted on a Vibratome (Leica VT1200) in a chamber filled with ice-cold artificial cerebrospinal fluid, ACSF, containing (in mM): 87 NaCl, 75 sucrose, 26 NaHCO_3_, 2.5 KCl, 1.25 NaH_2_PO_4_, 0.5 CaCl_2_, 7 MgSO_4_, 2.5 glucose, saturated with 95% O_2_, 5% CO_2_, pH 7.4. Transverse brain slices were cut at 400 μm thickness and kept at 35°C for 30 minutes. Slices were then cooled to room temperature and transferred to ACSF containing (in mM): 119 NaCl, 26 NaHCO_3_, 10 glucose, 2.5 KCl, 2.5 CaCl_2_, 1.3 MgCl_2_, 1 NaH_2_PO_4_. ACSF was equilibrated with 95% O_2_ and 5% CO_2_. The slices were stored in a submerged chamber for 1-7 h prior recording. In the submerged recording chamber, slices were perfused with ACSF at a rate of 3-4 ml/min at 34°C. Evoked postsynaptic were induced by stimulating Schaffer collaterals (0.1 Hz) in CA1 stratum radiatum with ACSF filled glass pipettes using a stimulus isolator (ISO-flex, A.M.P.I). Field excitatory postsynaptic potentials (fEPSPs) were recorded with glass pipettes filled with ACSF and placed in stratum radiatum. Stimulation artifacts were blanked in sample traces. Prolonged repetitive stimulation at 10 Hz was performed for 30 s in the presence of 50 μM APV (Tocris). LTP was induced by tetanic stimulation of Schaffer collaterals (two 1 s trains of 100 Hz, 20 s apart). Recordings were acquired with Axopatch 700B Amplifier (Molecular Devices), digitized at 5 kHz, filtered at 2 kHz, stored and analyzed using IGOR Pro 4 software (Wavemetrics). All data are expressed as mean ± SEM. Statistical significance for comparisons between groups was determined by unpaired t-tests.

### mEPSC recording

Whole-cell patch-clamp recordings of miniature excitatory postsynaptic currents (mEPSCs) have been described previously (Jüttner et al., 2005). Mice of postnatal day 24 to 28 were anaesthetized with isofluran and decapitated. The brain was quickly removed and placed in an ice-cold ACSF solution with a reduced calcium concentration consisting of (in mM): 130 NaCl, 4 KCl, 10 glucose, 1.25 NaH_2_PO_4_, 25 NaHCO_3_, 0.1 CaCl_2_, and 3 MgCl_2_. Transversal slices (200 µm) comprising the CA1 region of the hippocampus were obtained using a vibratome (VT1000S, Leica Microsystems, Germany) and were kept in oxygenated ACSF solution ((in mM) 130 NaCl, 4 KCl, 10 glucose, 1.25 NaH_2_PO_4_, 25 NaHCO_3_, 2 CaCl_2_, and 1 MgCl_2_) at room temperature for at least 2 hours. For subsequent patch-clamp recording, slices were transferred to a submersion-type recording chamber, which was continually perfused with oxygenated ACSF (2 ml/min). Miniature EPSCs were recorded from visually identified CA1 pyramidal neurons. AMPA-mediated mEPSCs were isolated pharmacologically by blocking glycinergic and GABAergic input as well as NMDA receptor-mediated currents (strychnine, 1 µM; bicuculline methiodide, 20 µM; APV, 100 µM). Action-potential dependent neurotransmitter release was blocked by TTX (1 µM). All experiments were performed at room temperature. During recordings slices were perfused at a flow rate of 2 ml/min with ACSF solution ((in mM) 130 NaCl, 4 KCl, 10 glucose, 1.25 NaH_2_PO_4_, 25 NaHCO_3_, 2 CaCl_2_, and 1 MgCl_2_). The patch pipette solution contained 4 mM NaCl, 120 mM KCl, 5 mM glucose, 5 mM EGTA, 10 mM HEPES, 0.5 mM CaCl_2_ and 4 mM MgCl_2_ (pH 7.3). During recordings at a holding potential of -70 mV the series resistance (10-20 MOhm) was electronically compensated as well as possible (50- 70%). Recordings were accepted only if the uncompensated series resistance was less than 20 MOhm and did not change by >20% during the experiment. All electrophysiological experiments were performed blinded for the genotype of the animals. Recordings were made using an EPC-10 (HEKA Electronics, Lambrecht/Pfalz, Germany). Signals were sampled at a rate of 10 kHz and analysed off-line using WinTida 5.0 (HEKA Electronics, Lambrecht/Pfalz, Germany). Postsynaptic events were analysed by PeakCount (C. Henneberger). The threshold for accepting events as mEPSCs was defined as 6 pA.

## Behavior and learning analysis

Barnes Maze analysis was performed as described previously ^11^. In brief, mice were trained for four days to find the hidden box positioned under the target hole on the underside of the Barnes maze. On day five, the box was closed. Number of pokes in each hole and the latency (time until reaching the target hole) were determined. Hole 1 to 9 were on the left side, hole -1 to -9 on the right side and (O) on the opposite (O) of the target hole (T).

Context-fear-conditioning was performed as previously described ^12^.

In a box with defined light setting and odor, a conditioned stimulus (CS, tone, 60 db, 10 kHz, 50 ms rise time) was applied twice for 15 s together with the unconditioned stimulus (US, electrical foot shock, 0,7 mA) after 13 s of the CS for 2 s. The next day mice were transferred back into the same box and observed for 3 minutes with the same light and odor settings as before. After 2 hours break, mice were transferred into a new box with different shape, light and odor. After three minutes the tone was applied for 3 min. The movement of mice during the conditioning, context and cue analysis was observed via the camera of the context-fear-conditioning setup and the percentage of freezing was calculated by the Med-Software (Med Associates).

The Open Field test was carried out in the native cage environment. The test apparatus (ActiMot, TSE systems) is a square-shaped frame with two pairs of light-beam strips, arranged at a right angle to determine the X and Y coordinates of the animal. Each mouse was placed individually into the middle of the cage and allowed to explore it freely for 3 min. For data analysis, the arena was divided in two areas, the periphery defined as a corridor along the walls and the remaining area representing the center of the arena. Statistical analysis was performed using unpaired-t test by GraphPad.

## Statistical analysis

For statistical analysis, GraphPad Prism 5.0 software was used. Results are expressed as means ± SEM. Statistical significance between groups was determined using the Mann Whitney U test for electrophysiology data. Expression values were compared using an unpaired two-tailed t test to assess differences between two groups. For comparison of more than one group the One-Way-ANOVA and for more than one condition the Two-Way-ANOVA was used. The significance level was chosen as p = 0.05.

# Supplementary References

1. Granseth, B., Odermatt, B., Royle, S. J. & Lagnado, L. Clathrin-mediated endocytosis is the dominant mechanism of vesicle retrieval at hippocampal synapses. *Neuron* **51**, 773–786 (2006).

2. Patzke, C. *et al.* The coxsackievirus-adenovirus receptor reveals complex homophilic and heterophilic interactions on neural cells. *J. Neurosci* **30**, 2897–2910 (2010).

3. Lisewski, U. *et al.* The tight junction protein CAR regulates cardiac conduction and cell-cell communication. *J Exp Med* **205**, 2369–79 (2008).

4. Bekkers, J. M. & Stevens, C. F. Excitatory and inhibitory autaptic currents in isolated hippocampal neurons maintained in cell culture. *Proc. Natl. Acad. Sci. U.S.A* **88**, 7834–7838 (1991).

5. Ronneberger, O., Fischer, P. & Brox, T. U-Net: Convolutional Networks for Biomedical Image Segmentation. *arXiv:1505.04597 [cs]* (2015).

6. Heinrich, L., Funke, J., Pape, C., Nunez-Iglesias, J. & Saalfeld, S. Synaptic Cleft Segmentation in Non-Isotropic Volume Electron Microscopy of the Complete Drosophila Brain. *arXiv:1805.02718 [cs]* (2018).

7. Roux, K. J., Kim, D. I., Raida, M. & Burke, B. A promiscuous biotin ligase fusion protein identifies proximal and interacting proteins in mammalian cells. *J. Cell Biol.* **196**, 801–810 (2012).

8. Stelzl, U. *et al.* A human protein-protein interaction network: a resource for annotating the proteome. *Cell* **122**, 957–968 (2005).

9. Hua, Y. *et al.* Blocking endocytosis enhances short-term synaptic depression under conditions of normal availability of vesicles. *Neuron* **80**, 343–349 (2013).

10. Schmitz, D., Mellor, J., Breustedt, J. & Nicoll, R. A. Presynaptic kainate receptors impart an associative property to hippocampal mossy fiber long-term potentiation. *Nat. Neurosci* **6**, 1058–1063 (2003).

11. Sunyer. Barnes maze, a useful task to assess spatial reference memory in the mice. *Nat Protoc* (2007). doi:10.1038/nprot.2007.390

12. Wehner, J. M. & Radcliffe, R. A. Cued and contextual fear conditioning in mice. *Curr Protoc Neurosci* **Chapter 8**, Unit 8.5C (2004).

# Supplementary figures

### Figure S1: CAR KO strategy.

CAR Exon1 is flanked by lox sites (dark triangles, floxed allele). Brain specific expression of the Cre recombinase under control of the CamKIIα- Promoter results in excision of exon 1 (deleted allele). Locations of the genotyping primers are indicated in the wildtype, floxed and deleted allele (black arrows). Exons (white box), frt sites (white triangle), lox sites (dark triangle).

### Figure S2: Rise time and decay time of mEPSCs in hippocampal slice as well as number of synapses in hippocampal cell culture are not altered.

**(A)** Examples traces for mEPSCs in control and CAR KO hippocampal slices (5-8 traces averaged). **(B, C)** Rise time (10-90%) and decay time constant of mEPSCs are not affected by the absence of CAR.

### Figure S3: Barnes Maze as a test for spatial memory.

(A) Learning phase (day 1 – 4) during the Barnes maze test of control and knockout mice. (B, C) Number of pokes into the target hole (T), holes 1 to 9 towards the left, holes -1 to -9 towards the right, and the opposite hole (O) are unchanged between KO and controls at day 5 (B) and day 10 (C) (CON, n = 18; KO, n = 17).

# Supplementary Table 1

| Symbol | | log2 Fold Change | | p-Value | |
| --- | --- | --- | --- | --- | --- |
| Cxadr | -3.549815 | | 3.31 10E-8 | |  |
| Slc6a7 | 1.097140 | | 0.000003 | |  |
| SNORA17 | 1.187963 | | 0.000056 | |  |
| Gm11291 | 0.851150 | | 0.000079 | |  |
| Gm6763 | 1.006353 | | 0.000080 | |  |
| Magea3 | 0.934750 | | 0.000102 | |  |
| Adh4 | -0.684675 | | 0.000141 | |  |
| Mir103-1 | 0.803987 | | 0.000153 | |  |
| Syt2 | 1.181323 | | 0.000220 | |  |
| 4921511M17Rik | 0.673523 | | 0.000283 | |  |
| Olfr637-ps1 | 0.915360 | | 0.000324 | |  |
| Gm6288 | -0.910013 | | 0.000325 | |  |
| Gm12257 | -0.608323 | | 0.000332 | |  |
| Gm7233 | -0.780410 | | 0.000383 | |  |
| Trbv12-2 | 0.780677 | | 0.000387 | |  |
| U6 | -0.582873 | | 0.000444 | |  |
| Olfr620 | 0.568050 | | 0.000494 | |  |
| Olfr1113 | -1.170953 | | 0.000513 | |  |
| Arsi | 0.560571 | | 0.000522 | |  |
| Gm11609 | 0.678507 | | 0.000531 | |  |
| Rpl30-ps5 | 0.731870 | | 0.000564 | |  |
| Nes | -0.538987 | | 0.000645 | |  |
| Acta2 | -0.631778 | | 0.000660 | |  |
| 4930594O21Rik | 0.645465 | | 0.000766 | |  |
| 2900073G15Rik | 0.689163 | | 0.000784 | |  |
| Olfr1292-ps1 | 0.554540 | | 0.000815 | |  |
| Slc46a2 | 0.593163 | | 0.000924 | |  |
| Olfr16 | -0.569155 | | 0.000935 | |  |
| Gm11717 | -1.036953 | | 0.000937 | |  |
| Vmn1r75 | 0.693692 | | 0.000973 | |  |
| Defb8 | -0.526550 | | 0.000979 | |  |
| Ighv12-3 | -0.519163 | | 0.001103 | |  |
| 2610024D14Rik | -1.200473 | | 0.001125 | |  |
| Ighv8-11 | 0.630677 | | 0.001170 | |  |
| Vmn1r201 | -0.653437 | | 0.001230 | |  |
| Olfr101 | -0.859617 | | 0.001266 | |  |
| Pmp2 | 0.671120 | | 0.001332 | |  |
| Gm11820 | -0.567317 | | 0.001346 | |  |
| Gm12264 | -0.671074 | | 0.001367 | |  |
| Olfr875 | 0.727090 | | 0.001377 | |  |
| Gm14936 | -0.452827 | | 0.001405 | |  |
| Pitx1 | 0.502410 | | 0.001535 | |  |
| Olfr125 | -0.598737 | | 0.001553 | |  |
| Abpg | 0.922800 | | 0.001578 | |  |
| Trim30a | -0.446432 | | 0.001584 | |  |
| 4930552N02Rik | -0.880287 | | 0.001586 | |  |
| Obox6 | -1.401400 | | 0.001612 | |  |
| Rps19-ps7 | -0.688483 | | 0.001670 | |  |
| Gm3776 | 0.438460 | | 0.001709 | |  |
| Omt2b | 0.695227 | | 0.001721 | |  |
| Olfr109 | 0.632710 | | 0.001744 | |  |
| Gm14466 | 0.641867 | | 0.001752 | |  |
| Olfr779 | -0.504350 | | 0.001769 | |  |
| Gm15663 | -0.853237 | | 0.001779 | |  |
| Mmp21 | 0.397026 | | 0.001798 | |  |
| Nudt6 | -0.665180 | | 0.001807 | |  |
| Olfr1368 | 0.568503 | | 0.001813 | |  |
| Olfr1032 | 1.057393 | | 0.001823 | |  |
| Gm14475 | -0.680475 | | 0.001831 | |  |
| Gm15218 | 0.754413 | | 0.001846 | |  |
| Klra8 | 0.695023 | | 0.001867 | |  |
| Tlr5 | -1.173490 | | 0.001917 | |  |
| Rab9 | -0.539321 | | 0.001927 | |  |
| Gm17032 | 0.574363 | | 0.001981 | |  |
| Gm15103 | 0.776000 | | 0.001988 | |  |
| Gm12298 | 0.403857 | | 0.002012 | |  |
| Trav6d-7 | 0.680200 | | 0.002051 | |  |
| F630206G17Rik | 0.528400 | | 0.002059 | |  |
| Clec2i | -0.639754 | | 0.002082 | |  |
| Tdrd6 | -0.545350 | | 0.002155 | |  |
| 1700006P19Rik | -1.052712 | | 0.002169 | |  |
| Olfr1447 | -0.921370 | | 0.002173 | |  |
| Gm15935 | 0.756010 | | 0.002431 | |  |
| Igkv15-103 | -0.403058 | | 0.002439 | |  |
| Olfr382 | -0.380425 | | 0.002477 | |  |
| Tmem126b | 0.648947 | | 0.002477 | |  |
| Gm12069 | 0.377423 | | 0.002566 | |  |
| Gm17007 | 0.612223 | | 0.002603 | |  |
| Gm14168 | -0.427820 | | 0.002704 | |  |
| Gm12839 | 0.500115 | | 0.002705 | |  |
| Txndc8 | -1.031150 | | 0.002717 | |  |
| Il2rg | -0.660407 | | 0.002723 | |  |
| Gm7749 | -0.554735 | | 0.002755 | |  |
| Gm5068 | -0.599657 | | 0.002791 | |  |
| Acnat1 | 0.426564 | | 0.002838 | |  |
| Olfr1321 | 0.458049 | | 0.002874 | |  |
| Hist2h2be | -0.724477 | | 0.002875 | |  |
| Olfr414 | 0.721270 | | 0.002977 | |  |
| Gm15867 | 0.383957 | | 0.003047 | |  |
| Gm16190 | 0.455406 | | 0.003054 | |  |
| F830016D02Rik | -0.607780 | | 0.003086 | |  |
| Vmn1r163 | 0.422622 | | 0.003095 | |  |
| Gm14007 | -0.661797 | | 0.003103 | |  |
| Gm14283 | -0.447677 | | 0.003111 | |  |
| Vmn1r227 | -0.475530 | | 0.003147 | |  |
| Gm14810 | 0.580663 | | 0.003149 | |  |
| Olfr94 | -0.627733 | | 0.003182 | |  |
| Pof1b | -0.571790 | | 0.003184 | |  |
| Gm12414 | 0.405457 | | 0.003217 | |  |
| Gm13103 | 0.409127 | | 0.003248 | |  |
| Gm11959 | -0.390906 | | 0.003363 | |  |
| Gm53 | 0.499557 | | 0.003384 | |  |
| 4930556N08Rik | 0.478023 | | 0.003460 | |  |
| 1700080N15Rik | -0.389284 | | 0.003550 | |  |
| Gm16555 | 0.444767 | | 0.003559 | |  |
| Gm13924 | 1.332330 | | 0.003563 | |  |
| Olfr9 | 0.599323 | | 0.003590 | |  |
| Vmn1r76 | 0.629513 | | 0.003606 | |  |
| Relb | 0.607290 | | 0.003627 | |  |
| Olfr1206 | -0.682357 | | 0.003725 | |  |
| Gm6457 | -0.507619 | | 0.003799 | |  |
| Olfr1469 | 0.535060 | | 0.003816 | |  |
| Gm16884 | 0.377814 | | 0.003852 | |  |
| Dennd2d | 0.533320 | | 0.003914 | |  |
| Sectm1b | 0.736827 | | 0.003951 | |  |
| Gm17549 | -0.702698 | | 0.004000 | |  |
| Rdh13 | 0.336647 | | 0.004041 | |  |
| Dmgdh | 0.691897 | | 0.004057 | |  |
| Parp9 | 0.847093 | | 0.004104 | |  |
| Mrgpra3 | 0.389101 | | 0.004105 | |  |
| Gm12673 | 0.372583 | | 0.004152 | |  |
| Eln | -0.363429 | | 0.004181 | |  |
| Gm14217 | -0.859160 | | 0.004184 | |  |
| Tas2r119 | 0.465217 | | 0.004197 | |  |
| Il18r1 | 0.431653 | | 0.004235 | |  |
| Gm11286 | 0.955380 | | 0.004275 | |  |
| Gm13874 | 0.621862 | | 0.004277 | |  |
| Gm16656 | 0.631810 | | 0.004288 | |  |
| Gm13893 | 0.950388 | | 0.004316 | |  |
| Gm12617 | 0.538053 | | 0.004324 | |  |
| Gm12649 | 1.004547 | | 0.004328 | |  |
| Fam92b | 0.379793 | | 0.004330 | |  |
| Gm11678 | -0.421227 | | 0.004353 | |  |
| 1700018A04Rik | 0.523428 | | 0.004407 | |  |
| Timp1 | -0.378673 | | 0.004414 | |  |
| Gm13219 | 0.413917 | | 0.004501 | |  |
| 3110009E18Rik | -0.527310 | | 0.004573 | |  |
| A930017K11Rik | 0.430229 | | 0.004613 | |  |
| Gm14671 | -0.480735 | | 0.004616 | |  |
| A630033H20Rik | -0.428559 | | 0.004645 | |  |
| Rpl10-ps4 | 0.731610 | | 0.004686 | |  |
| Sp140 | -0.633468 | | 0.004691 | |  |
| Gm9999 | -0.438928 | | 0.004698 | |  |
| Olfr760-ps1 | -0.420213 | | 0.004734 | |  |
| Gm8805 | -0.862753 | | 0.004795 | |  |
| Gm13070 | -0.402297 | | 0.004883 | |  |
| Gm12376 | 0.425505 | | 0.004923 | |  |
| 2310003L06Rik | 1.300647 | | 0.004954 | |  |
| Cdkl1 | -0.386691 | | 0.005042 | |  |
| 4930483K19Rik | 0.520482 | | 0.005092 | |  |
| Gm12813 | 0.711445 | | 0.005197 | |  |
| Car6 | 0.347012 | | 0.005209 | |  |
| Ikzf1 | 0.470203 | | 0.005251 | |  |
| Osm | 0.531452 | | 0.005300 | |  |
| Pstk | -0.519675 | | 0.005458 | |  |
| Olfr123 | 0.601700 | | 0.005469 | |  |
| Gm6378 | -0.748483 | | 0.005493 | |  |
| Fam83f | 0.355883 | | 0.005546 | |  |
| Osgin2 | -0.408903 | | 0.005602 | |  |
| 4921525B02Rik | -0.638735 | | 0.005612 | |  |
| Olfr1063-ps1 | 0.465193 | | 0.005628 | |  |
| Gm10439 | 0.491465 | | 0.005632 | |  |
| Gm5093 | -0.691043 | | 0.005639 | |  |
| Myh11 | -0.336168 | | 0.005656 | |  |
| Gm17227 | 0.505637 | | 0.005706 | |  |
| Ammecr1 | -0.465292 | | 0.005790 | |  |
| Gm15362 | 0.565538 | | 0.005858 | |  |
| Gm16325 | 0.699150 | | 0.005860 | |  |
| Gm2529 | 0.419922 | | 0.005890 | |  |
| Gm17598 | 0.492843 | | 0.005899 | |  |
| Gjc2 | 0.403310 | | 0.005923 | |  |
| Gm16267 | 0.556377 | | 0.005939 | |  |
| Arl6ip6 | -0.438712 | | 0.005940 | |  |
| Rdh18-ps | 0.443883 | | 0.005949 | |  |
| Gm9060 | 0.569833 | | 0.005952 | |  |
| Vmn1r30 | 0.530057 | | 0.005961 | |  |
| Gm15917 | 0.477583 | | 0.005992 | |  |
| Olfr1110 | 1.365697 | | 0.006016 | |  |
| Hoxa3 | 0.445640 | | 0.006022 | |  |
| 2900093L17Rik | -0.371802 | | 0.006041 | |  |
| Gm15345 | 0.395105 | | 0.006099 | |  |
| Pop7 | -0.495103 | | 0.006100 | |  |
| Cyp2c50 | -0.492743 | | 0.006181 | |  |
| Gm10345 | 0.446168 | | 0.006226 | |  |
| Hormad2 | 0.549779 | | 0.006230 | |  |
| Gm15534 | -0.350234 | | 0.006268 | |  |
| Gm12901 | 0.548260 | | 0.006281 | |  |
| Gm16322 | -0.399568 | | 0.006298 | |  |
| Gm15690 | 0.498647 | | 0.006336 | |  |
| Heyl | -0.455669 | | 0.006399 | |  |
| Gm5757 | -0.442708 | | 0.006400 | |  |
| Gm14964 | -0.516128 | | 0.006433 | |  |
| Gm12429 | 0.565005 | | 0.006475 | |  |
| 1700080G11Rik | 0.723397 | | 0.006500 | |  |
| 1700020C07Rik | -0.652017 | | 0.006550 | |  |
| Gm10528 | 0.450044 | | 0.006556 | |  |
| Mup-ps16 | 0.512480 | | 0.006618 | |  |
| A330043C09Rik | 0.691497 | | 0.006621 | |  |
| Apol7d | 0.382017 | | 0.006701 | |  |
| Gm16538 | 0.415953 | | 0.006740 | |  |
| Pxmp3 | -0.342283 | | 0.006772 | |  |
| G630030J09Rik | 0.397097 | | 0.006773 | |  |
| Fhl5 | -0.445230 | | 0.006816 | |  |
| Gm5531 | 0.435973 | | 0.006845 | |  |
| Ms4a4b | 0.384410 | | 0.006865 | |  |
| Spint4 | 0.855953 | | 0.006869 | |  |
| Vmn1r17 | -0.418732 | | 0.006919 | |  |
| Gm15920 | 0.400403 | | 0.006980 | |  |
| V1ra8 | -0.391307 | | 0.007055 | |  |
| Gm9979 | -0.342102 | | 0.007135 | |  |
| Olfr1150-ps1 | 0.539405 | | 0.007238 | |  |
| Tagln | -0.339335 | | 0.007306 | |  |
| Gm13559 | -0.684613 | | 0.007323 | |  |
| Abcb4 | -0.847573 | | 0.007362 | |  |
| Klra4 | 0.474072 | | 0.007503 | |  |
| Xkrx | 0.469063 | | 0.007606 | |  |
| Gm12766 | 0.342691 | | 0.007646 | |  |
| Gm14772 | 0.308472 | | 0.007681 | |  |
| Xlr | -0.661093 | | 0.007744 | |  |
| Gm14753 | -0.326199 | | 0.007754 | |  |
| Tssk4 | 0.399291 | | 0.007768 | |  |
| Clec3a | 0.536687 | | 0.007771 | |  |
| Gm7958 | 0.489681 | | 0.007805 | |  |
| Tmem106a | 0.291188 | | 0.007874 | |  |
| Olfr616 | 0.395753 | | 0.007886 | |  |
| Gm10397 | -0.505072 | | 0.007888 | |  |
| Spats2l | -0.544790 | | 0.007899 | |  |
| Olfr146 | 0.568817 | | 0.007941 | |  |
| Map3k7 | -0.518073 | | 0.008004 | |  |
| Nlrp9c | 0.520640 | | 0.008010 | |  |
| H2-Q7 | -0.817537 | | 0.008083 | |  |
| Gm10923 | 0.619857 | | 0.008091 | |  |
| Gm16219 | -0.674117 | | 0.008146 | |  |
| Gm16086 | -0.559510 | | 0.008150 | |  |
| Gpr84 | -0.311453 | | 0.008299 | |  |
| Efcab9 | -0.597252 | | 0.008301 | |  |
| Gm14752 | -0.833328 | | 0.008314 | |  |
| Gm13274 | 0.411297 | | 0.008325 | |  |
| Mir143 | 0.754713 | | 0.008382 | |  |
| Gm16123 | 0.761223 | | 0.008392 | |  |
| Gm8312 | 0.453507 | | 0.008407 | |  |
| Gm13688 | -0.438203 | | 0.008446 | |  |
| Gm14925 | 0.800893 | | 0.008478 | |  |
| 2310009B15Rik | -0.493552 | | 0.008580 | |  |
| Gm4794 | 0.503322 | | 0.008604 | |  |
| C130079G13Rik | 0.354261 | | 0.008649 | |  |
| Gsg2 | -0.350823 | | 0.008706 | |  |
| 1700030A11Rik | -0.389410 | | 0.008729 | |  |
| Olfr808 | 0.482264 | | 0.008731 | |  |
| Gm6664 | 0.513646 | | 0.008763 | |  |
| Fam159a | 0.576723 | | 0.008843 | |  |
| Gm17724 | 0.303093 | | 0.008844 | |  |
| H3f3a-ps1 | -0.630100 | | 0.008873 | |  |
| Rcc1 | -0.454463 | | 0.008901 | |  |
| Olfr1179 | 0.363488 | | 0.008935 | |  |
| Acmsd | -0.436910 | | 0.008986 | |  |
| Gm12961 | -0.418313 | | 0.009010 | |  |
| Lcn6 | 0.344983 | | 0.009097 | |  |
| Olfr1217 | 0.605852 | | 0.009114 | |  |
| Il1f8 | 0.342774 | | 0.009117 | |  |
| Gm13706 | -0.396290 | | 0.009125 | |  |
| Olfr591 | 0.479098 | | 0.009151 | |  |
| Olfr761 | 0.381383 | | 0.009169 | |  |
| Olfr370 | 0.406801 | | 0.009252 | |  |
| Gm11579 | -0.406048 | | 0.009364 | |  |
| Ppyr1 | -0.331303 | | 0.009457 | |  |
| Gm13041 | 0.293763 | | 0.009500 | |  |
| Gm15318 | 0.446937 | | 0.009531 | |  |
| Gm12698 | 0.550250 | | 0.009547 | |  |
| Sypl | -0.345396 | | 0.009597 | |  |
| Olfr488 | -0.425153 | | 0.009676 | |  |
| Krcc1 | -0.404725 | | 0.009699 | |  |
| Gm4912 | 0.378990 | | 0.009802 | |  |
| Fam98c | 0.423427 | | 0.009830 | |  |
| Gm5930 | 0.516197 | | 0.009878 | |  |
| Gm16734 | 0.421392 | | 0.009916 | |  |
| Gm3511 | -0.525372 | | 0.009958 | |  |
| Gm2343 | 0.642347 | | 0.009964 | |  |
| AA467197 | 0.290014 | | 0.009973 | |  |
| Ube2u | -0.390018 | | 0.009978 | |  |

**Supplementary table 2.** Proteins binding to the CAR cytoplasmic domain (Yeast-Two-Hybrid screen).

| **Gene_id** | **Symbol** | **GO-term** | **SDIV-repeats** |
| --- | --- | --- | --- |
| 222484 | LNX2 | cell adhesion | 4 |
| 1742 | PSD95 | synaptic transmission | 4 |
| 6386 | SDCBP | synaptic transmission | 4 |
| 80149 | ZC3H12A | regulation of gene expression | 4 |
| 84709 | C4orf49 | - | 3 |
| 10241 | Calcoco2 | viral reproduction | 2 |
| 27338 | UBE2S | metabolic process | 2 |
| 5888 | RAD51 | DNA repair | 2 |
| 3512 | IGJ | immune response | 1 |
| 6569 | SLC34A1 | transmembrane transport | 1 |
| 11156 | PTP4A3 | dephorphorylation | 1 |
| 26286 | ARFGAP3 | protein transport | 1 |
| 60482 | SLC5A7 | synaptic transmission | 1 |
| 1453 | CSNK1D | protein phosphorylation | 1 |
| 64840 | PORCN | glycoprotein metabolic process | 1 |
| 9118 | INA | neurofilament cytosceleton organization | 1 |
| 960 | CD44 | cell adhesion | 1 |
| 91607 | SLFN11 | - | 1 |
| 23047 | PDS5B | cell proliferation | 1 |
| 54541 | DDIT4 | cell proliferation | 1 |
| 6988 | TCTA | - | 1 |
| 4714 | NDUFB8 | electron transport chain | 1 |
| 3818 | KLKB1 | extracellular matrix organization | 1 |
| 5657 | PRTN3 | cell proliferation | 1 |
| 64968 | MRPS6 | translation | 1 |
| 57456 | KIAA1143 | - | 1 |
| 4597 | MVD | metabolic process | 1 |
| 6461 | SHB | cell proliferation | 1 |

**Supplementary table 3.** Biotinylated proteins identified with the BioID proximity assay using the CAR-BirA* fusion-protein.

| **Uniprot** | **Symbol** | **GO-term** | **PEP** | **Intensity control** | **Intensity CAR-BirA*** |
| --- | --- | --- | --- | --- | --- |
| Q9R066 | Cxadr | cell adhesion | 7.76E-133 | 0 | 1,662,800,000 |
| Q9Z270 | Vapa | synaptic transmission | 3.69E-41 | 0 | 30,593,000 |
| D3ZM03 | RGD1566155 | - | 5.86E-27 | 0 | 17,905,000 |
| Q5PQX1 | Tor1aip1 | nuclear envelope organization | 5.90E-22 | 0 | 13,971,000 |
| Q5XIU9 | Pgrmc2 | - | 2.61E-09 | 0 | 148,520,000 |
| Q4KM74 | Sec22b | protein transport | 1.34E-07 | 0 | 61,441,000 |
| Q62733 | Tmpo | nuclear envelope organization | 2.49E-07 | 0 | 32,922,000 |
| P06762 | Hmox1 | metabolic process | 7.01E-07 | 0 | 40,983,000 |
| P70580 | Pgrmc1 | axon guidance | 7.28E-07 | 0 | 43,731,000 |
| Q4FZX7 | Srprb | protein transport | 5.01E-05 | 0 | 34,863,000 |
| O35112 | Alcam | cell adhesion | 1.45E-04 | 0 | 262,030,000 |
| Q9Z269 | Vapb | synaptic transmission | 3.99E-04 | 0 | 26,219,000 |
| Q6AY18 | Sar1a | protein transport | 4.24E-04 | 0 | 8,464,000 |
| Q4QRB4 | Tubb3 | axon guidance | 4.97E-04 | 0 | 8,045,800 |
| B1WC88 | CR032 | - | 2.84E-03 | 0 | 65,639,000 |
| Q9WUW2 | Vamp2 | synaptic transmission | 4.18E-03 | 0 | 444,470,000 |
| Q7TP42 | Sec62 | protein transport | 5.33E-03 | 0 | 17,596,000 |
| Q62896 | Bet1 | protein transport | 8.87E-03 | 0 | 18,898,000 |
| P30839 | Aldh3a2 | metabolic process | 1.12E-02 | 0 | 24,258,000 |
| B2RZD1 | Sec61b | protein transport | 1.15E-02 | 0 | 118,330,000 |
| Q5RJR8 | Lrrc59 | - | 1.28E-02 | 0 | 40,267,000 |
| Q6AYT7 | Abd12 | - | 1.46E-02 | 0 | 26,160,000 |
| Q5BK13 | Tmem199 | - | 2.13E-02 | 0 | 14,637,000 |
